# Characterization of self-emulsifying macadamia nut oil fermented by *Epidermidibacterium keratini* mutant EPI-7-i originated from skin flora as a novel cosmetic ingredient

**DOI:** 10.1038/s41598-026-47367-z

**Published:** 2026-04-09

**Authors:** Hyo-Bin Kim, Shin-Joung Rho, Hyeok Nam-gung, Hui Beom Park, Seok Kyun Yun, Juhyun Son, Ju Hun Lee, Hyung Hee Baek, Seunghyun Kang, Sugyeong Jeong, Yong-Ro Kim

**Affiliations:** 1 Department of Biosystems Engineering, Seoul National University, 1, Gwanak-ro, Gwanak-gu, Seoul, 08826 Republic of Korea; 2 Convergence Major in Global Smart Farm, Seoul National University, Seoul, 08826 Republic of Korea; 3 Center for Food and Bioconvergence, Seoul National University, Seoul, 08826 Republic of Korea; 4 COSMAX BTI, 255, Pangyo-ro, Bundang-gu, Seongnam, 13486 Republic of Korea; 5 COSMAX AB, Seoul, 08390 Republic of Korea; 6 Department of Food Engineering, Dankook University, Cheonan, 31116 Republic of Korea; 7 Research Institute of Agriculture and Life Sciences, Seoul National University, Seoul, 08826 Republic of Korea

**Keywords:** *Epidermidibacterium keratini*, Macadamia nut oil, Microbial bioconversion, Self-emulsifying oil, Biosurfactant lipids, Nanoemulsion stability, Biochemistry, Biological techniques, Biotechnology, Chemistry, Microbiology

## Abstract

Microbial bioconversion has emerged as a sustainable strategy to replace petroleum-based surfactants; however, its application in generating self-emulsifying oils for cosmetic formulations remains limited. This study aimed to develop self-emulsifying oils derived from macadamia nut oil (MNO) through microbial lipid remodeling and to evaluate their physicochemical and functional properties. A skin-derived bacterium, *Epidermidibacterium keratini* mutant (EPI-7-i), was employed to bioconvert MNO for 2 days (MNO-M1) and 5 days (MNO-M2), after which the self-emulsifying oils were recovered as solvent-extracted lipid fractions from the fermentation system, rather than from the whole culture broth. Lipid composition and emulsion characteristics were analyzed using UHPLC–MS/MS and physicochemical stability assessments. Bioconversion induced extensive lipid remodeling, yielding oils enriched in monoacylglycerols (MAG), diacylglycerols (DAG), free fatty acids (FA), and ether-linked glycerides (alkylacylglycerols, DG O). These compositional changes enabled spontaneous formation of oil-in-water (O/W) nanoemulsions without external surfactants. The emulsions exhibited nanoscale droplet sizes (~ 260 nm), high absolute ζ-potential values (> 45 mV), and stability against thermal and pH stress over 28 days. Despite their high unsaturated lipid content, bioconverted oil emulsions displayed enhanced oxidative stability compared with emulsions prepared from enzymatically modified oil (MNO-E), which was attributed to the formation of microbially derived antioxidant metabolites, including ether lipids structurally analogous to plasmalogens. These results demonstrate that microbial bioconversion enables the integration of emulsification and antioxidative functions within a single oil phase and suggest the potential of solvent-extracted self-emulsifying oils as sustainable, clean-label alternatives for cosmetic nanoemulsion systems.

**Supplementary Information:**

The online version contains supplementary material available at 10.1038/s41598-026-47367-z.

## Introduction

Surfactants are amphiphilic substances that reduce surface and interfacial tension, thereby enabling emulsification, dispersion, wetting, and foaming—functions that are essential across the food, pharmaceutical, agricultural, and cosmetic industries^1,2^. Despite their broad utility, most commercial surfactants are derived from petroleum-derived feedstocks and are associated with environmental persistence and potential toxicity. In response to increasing regulatory and consumer demand for sustainable and skin-compatible ingredients, biosurfactants produced by microorganisms from renewable resources have gained considerable attention as eco-friendly alternatives^1,2^. Compared with synthetic surfactants, biosurfactants are typically biodegradable, exhibit low toxicity, and possess high surface activity under mild conditions, making them attractive candidates for next-generation formulations^3^. Due to these advantages, their applications have expanded to include enhanced oil recovery, wastewater treatment, agriculture, pharmaceuticals, food systems, detergents, and cosmetic products^3,4^.

In cosmetic formulations, surfactant selection is particularly critical due to direct and prolonged skin contact. Conventional chemical surfactants have been reported to disrupt the stratum corneum lipid matrix and alter the skin microbiome, potentially leading to irritation, barrier dysfunction, and adverse immunological responses^5,6^. As a result, naturally derived and biologically compatible surfactants have emerged as preferred alternatives. Microbial glycolipid biosurfactants such as sophorolipids, rhamnolipids, and mannosylerythritol lipids have been incorporated into a range of personal care products, including cleansers, shampoos, creams, and lotions, owing to their favorable interfacial properties and additional functionalities such as moisturizing, antimicrobial, and anti-inflammatory effects^7^.

Despite these advances, systematic investigations into the emulsification and stabilization mechanisms of biosurfactant-based systems remain limited, particularly for cosmetic emulsions^8^. Emulsions are inherently thermodynamically unstable and susceptible to physical destabilization processes such as coalescence, flocculation, and Ostwald ripening^9^. Their kinetic stability is governed by interfacial composition, droplet charge, and molecular interactions at the oil–water interface, all of which are strongly influenced by the nature of the emulsifier^10,11^. Moreover, emulsified systems are especially vulnerable to lipid oxidation due to increased interfacial areas and the proximity of pro-oxidant species, which can compromise product performance, safety, and shelf life^12^. Therefore, developing emulsifiers that simultaneously provide interfacial stabilization and oxidative protection remains a major challenge in cosmetic formulation science.

Microbial fermentation has emerged as a versatile platform for producing multifunctional cosmetic ingredients with improved sustainability and bioactivity^13,14^. Microbial metabolites, including polysaccharides, peptides, enzymes, antioxidants, pigments, and biosurfactants, exhibit a wide range of skin-beneficial effects such as antioxidative, anti-aging, and antimicrobial activities^13,14^. Among the diverse metabolites produced during microbial fermentation, lipid-derived compounds have recently attracted attention due to their potential roles in interfacial activity and emulsion stabilization. Among these, ether-linked glycerides, such as alkylacylglycerols (DG O), are of particular interest. Ether-linked glycerides are known to contain an ether bond at the sn-1 position of the glycerol backbone. Structurally related ether lipids, particularly those containing vinyl ether linkages (e.g., plasmalogens), are known to contribute to membrane stabilization and antioxidant defense in biological systems^15,16^. Of particular relevance are metabolites derived from skin-resident microorganisms, which are associated with maintaining skin homeostasis. For instance, fatty acids and glycerol produced by commensal bacteria contribute to barrier integrity, hydration, and microbiome balance^17,18^.

*Epidermidibacterium keratini* (EPI-7), a bacterial species isolated from human skin, has recently been identified as a promising probiotic candidate for dermatological applications^19^. Previous studies have shown that its metabolites can modulate the skin microbiome and exert anti-aging and protective effects^19–21^. However, the capacity of *E. keratini* to remodel lipids and generate interfacially active compounds suitable for emulsion-based formulations has not yet been explored.

In this study, a mutant strain of *E. keratini* (EPI-7-i) was employed to bioconvert macadamia nut oil (MNO) into an intrinsically self-emulsifying oil. MNO was selected not only for its well-established skin compatibility but also for its favorable lipid composition, particularly its high oleic acid content. Oleic acid, the predominant fatty acid in MNO, is readily utilized by various microorganisms as a carbon source and can support microbial growth and biotransformation processes via β-oxidation and related metabolic pathways^22^. In addition, the lipid composition of MNO, which is rich in neutral lipids such as triacylglycerols, resembles sebum-derived substrates naturally available to skin-associated microorganisms. Skin-resident bacteria are known to metabolize lipid-rich substrates, including triglycerides, through lipolytic activity^23,24^. Therefore, MNO provides a metabolically favorable and biologically relevant substrate for microbial lipid remodeling. Furthermore, its high content of monounsaturated fatty acids has been associated with beneficial dermatological properties, including improved skin hydration and anti-aging effects^25,26^. These combined physicochemical and biological characteristics make MNO an attractive substrate for microbial bioconversion aimed at producing value-added lipid-based materials. The study was based on the hypothesis that microbial bioconversion induces lipid remodeling, including lipolysis and the formation of structurally diverse lipid species. This process transforms triacylglycerol-rich MNO into a functionally enhanced oil enriched with endogenous biosurfactants and antioxidant metabolites. The physicochemical properties of the resulting self-emulsifying oil were characterized, and the emulsification behavior, interfacial properties, and oxidative stability of nanoemulsions prepared from the bioconverted oil were evaluated and compared with those of enzymatically modified MNO (MNO-E). This work presents a microbial bioconversion–based approach to producing self-emulsifying oils with improved oxidative stability. The resulting oils integrate emulsifying and antioxidant functions and demonstrate potential as a sustainable alternative to petroleum-based surfactants for cosmetic nanoemulsions.

## Materials and methods

### Materials

*Epidermidibacterium keratini* mutant (EPI-7-i) was provided by COSMAX AB, Inc. (Seoul, Korea). Macadamia nut oil (MNO) was purchased from Oleon Ltd. (Northallerton, North Yorkshire, UK). *Candida antarctica* lipase B immobilized on Immobead 150 (≥ 2000 U/g; Sigma-Aldrich, St. Louis, MO, USA) was used for enzymatic modification. 2,2-Diphenyl‐1‐picrylhydrazyl (DPPH), glycerol, *n*‐hexane, ethanol, sodium azide, oleic acid, dodecanedioic acid, Nile red, *n*‐butanol, ammonium thiocyanate, ferrous sulfate, cumene hydroperoxide, and α-tocopherol were purchased from Sigma‐Aldrich (St. Louis, MO, USA). Methanol, chloroform, HCl, and NaOH were purchased from Duksan Pure Chemicals Co., Ltd. (Ansan, Korea). Cyclo(Phe-Pro) and (Z)-9,10-dihydroxyoctadec-12-enoic acid were obtained from Cayman Chemical Co. (Ann Arbor, MI, USA). Isooctane and 2‐propanol were purchased from Samchun Pure Chemicals Co., Ltd. (Seoul, Korea).

### Production of self-emulsifying oil

#### Production of microbially converted macadamia nut oil (MNO-M)

The strain EPI-7-i was initially cultivated in a preculture medium containing MNO at 30 °C with agitation at 180 rpm for 18 h. Subsequently, 10% (v/v) of the preculture was inoculated into a 5.5 L fermenter containing 3.0 L of main culture medium and incubated at 30 °C and 180 rpm for 2 or 5 days. The preculture medium consisted of 50 mL/L MNO, 0.1 g/L papain digest soybean, 1.0 g/L yeast extract, 1.0 g/L glycerin, 4.5 g/L KH_2_PO_4_, and 3.0 g/L K_2_HPO_4_. The main culture medium contained 100 mL/L MNO, 0.1 g/L papain digest soybean, 1.0 g/L yeast extract, 0.1 g/L glycerin, 4.5 g/L KH_2_PO_4_, 3.0 g/L K_2_HPO_4_, 1.0 g/L (NH_4_)_2_SO_4_, 0.5 g/L MgSO_4_∙7H_2_O, 0.1 g/L KNO_3_, and 0.3 g/L CaCl_2_. After fermentation, the cultures were centrifuged at 5000 × *g* for 10 min (Supra 22 K, Hanil Science Inc., Incheon, Korea) to obtain cell-free supernatants, and the pH was adjusted to 2.0–3.0 using 2 mol/L HCl. To obtain the microbially transformed oil fraction distinct from the original MNO, the acidified supernatants were subjected to liquid-liquid extraction using a chloroform: methanol (1:1, v/v) mixture at a solvent-to-supernatant ratio of 2:1 (v/v). The organic phase was collected, and the solvent was removed using a rotary evaporator (Sunil Eyela Ltd., Seongnam, Korea). The oils extracted from cultures fermented for 2 and 5 days were designated as MNO-M1 and MNO-M2, respectively. MNO-M is officially registered as “*Epidermidibacterium Keratini*/Macadamia Ternifolia Seed Oil Ferment Extract Filtrate” in the Personal Care Products Council database (https://www.personalcarecouncil.org/).

#### Production of enzymatically converted macadamia nut oil (MNO-E)

For comparison of emulsifying properties with MNO-M, enzymatically converted macadamia nut oil (MNO-E) was prepared using *Candida antarctica* lipase B, following a previously reported method^27^. Briefly, 30 g of MNO was placed in 100 mL glass bottle and mixed with glycerol at a 1:1 molar ratio relative to the triacylglycerol (TAG) content. The required glycerol amount was calculated based on its molecular weight (92 g/mol) and the estimated TAG molecular weight, which was determined as three times 56,000 divided by the saponification value (SV) of the oil. Deionized water equivalent to 3.5% (w/w) of the glycerol mass was added to the reaction mixture. Immobilized *Candida antarctica* lipase B (non-regiospecific, immobead 150) was incorporated at 2% (w/w) of the oil. After gentle agitation to achieve uniform enzyme dispersion, the reaction mixture was incubated in water bath at 65 °C with shaking at 130 rpm for 48 h. The mixture was then centrifuged at 495 × g for 5 min to separate MNO-E from the immobilized enzyme, residual glycerol, and water.

### Fractionation of self-emulsifying oils

Prior to UHPLC–MS/MS analysis, all oil samples (MNO, MNO-M, and MNO-E) were subjected to solvent fractionation to separate and compare the relatively nonpolar and polar lipid components. The fractionation of MNO and MNO-M was performed based on the method reported by Montealegre et al.^28^. Fifty grams of oil were dissolved in 200 mL of *n*‐hexane, after which the solution was extracted three times with 50 mL portions of an ethanol: water mixture (87:13, v/v). Additional ethanol-soluble fractions were recovered by sequentially adding 50 mL of *n*‐hexane to the remaining hexane layer until no further phase separation occurred. The combined ethanolic fractions were washed once with 50 mL of *n*‐hexane, and the oils were isolated by vacuum evaporation of the solvent. The dried weights of the fractionated oils were measured by a gravimetric method. Fractionation of MNO‐E was conducted based on the procedure reported by Zhang et al.^29^, with slight modifications. Briefly, 7 g of MNO‐E were dissolved in 210 mL of *n*‐hexane and 210 mL of 87% (v/v) ethanol. After phase separation, the aqueous ethanol layer was collected and washed once with 210 mL of *n*‐hexane. The fractionated oils were recovered by evaporating the solvent under vacuum, and the dried weights were recorded gravimetrically.

### UHPLC-MS/MS analysis

The chemical composition of oil samples fractionated using hexane and ethanol solvents was determined by ultrahigh-performance liquid chromatography combined with tandem mass spectrometry (UHPLC-MS/MS)^30^. Prior to the analysis, the fractionated oils were diluted at ratios of 1:100 or 1:1000 in a chloroform–methanol mixture (50:50, v/v). Chromatographic separation was performed using a Dionex Ultimate 3000 UHPLC system (Thermo Fisher Scientific, MA, USA) fitted with an Acquity UPLC BEH C_18_ column (100 mm × 2.1 mm, 1.7 μm) maintained at 45 °C. The mobile phase consisted of solvent A (acetonitrile–water, 60:40, v/v) and solvent B (isopropanol–acetonitrile, 90:10, v/v), both containing 10 mmol/L ammonium formate and 0.1% (v/v) acetic acid. The flow rate was set at 0.25 mL/min, and the proportion of solvent A was linearly decreased from 95% to 0% over 23 min, followed by 0% A for 2 min, then returned to 95% A for re-equilibration.

Mass spectrometric analysis was carried out on a Triple TOF 5600 + instrument (AB SCIEX, Marlborough, MA, USA) with electrospray ionization (ESI) in both positive and negative ion modes. Source conditions were: spray voltage + 5500 V (positive) and − 4500 V (negative), source temperature 500 °C, nebulizer and heater gas at 50 psi, and curtain gas at 25 psi. Data were acquired in full-scan mode over an *m/z* range of 100–2000.

Lipid species were identified based on molecular masses, retention time, and MS/MS fragmentation patterns using Scaffold Elements (version 2.1.1, Proteome Software, Inc., Portland, OR, USA; https://www.proteomesoftware.com/products/scaffold-elements). Quantification was performed using PeakView (version 2.2, AB Sciex, Foster City, CA, USA; https://sciex.com/products/software/peakview-software) and MultiQuant (version 3.0.3, AB Sciex, Foster City, CA, USA; https://sciex.com/products/software/multiquant-software) with calibration curves established using surrogate standards^31^.

Due to the limited commercial availability of authentic standards for alkylacylglycerols (DG O), their concentrations were estimated using structurally similar diacylglycerol (DAG) standards as analog internal standards, based on similarities in retention time, ionization behavior, and molecular structure. This approach provides a semi-quantitative estimation rather than absolute quantification and may introduce some degree of uncertainty due to differences in ionization efficiency between DG O and DAG. To address instrumental variations and matrix interferences, internal standards were used, with details provided in Table S1.

### Volatile compound analysis

Volatile compounds in the self-emulsifying oil samples were extracted using headspace solid-phase microextraction (HS-SPME) with a 50/30 µm DVB/CAR/PDMS fiber (Supelco Co., Bellefonte, PA, USA)^32^. Four milliliters of each oil sample (MNO, MNO-M1, and MNO-M2) were transferred into a sealed headspace vial, equilibrated at 40 °C for 30 min, and then exposed to the SPME fiber for 30 min for volatile adsorption. The fiber was subsequently desorbed in the GC injector at 200 °C for 10 min.

Volatile profiles were analyzed using a gas chromatography–mass spectrometry (GC–MS) system (Agilent 7890B GC coupled to a 5977 A mass selective detector, Agilent Technologies, Palo Alto, CA, USA) with a DB-WAX capillary column (60 m × 0.25 mm, 0.25 μm; J&W Scientific, Folsom, CA, USA). The oven program was 40 °C for 5 min, ramped to 200 °C at 5 °C/min, and held at 200 °C for 20 min. Helium served as the carrier gas at 1.0 mL/min. Mass spectrometry was performed with electron ionization at 70 eV, scanning *m/z* 33–350. Volatiles were identified by comparing mass spectra and retention indices with the Wiley 9th Edition/NIST08 mass spectral library.

### Antioxidant activity of self-emulsifying oil

The antioxidant capacity of the self-emulsifying oils was determined by 2,2-diphenyl-1-picrylhydrazyl (DPPH) radical scavenging assay^33^. Each self-emulsifying oil sample (10 µL) was mixed with 990 µL of 0.07 mmol/L DPPH solution prepared in ethanol. After vortexing, the mixture was incubated in the dark for 30 min. Absorbance was measured at 515 nm using a UV-Vis spectrophotometer (UV-1650PC, Shimadzu, Kyoto, Japan). Ethanol was used in place of the DPPH solution for blank measurements, while the control consisted of ethanol replacing the oil sample. To quantify radical scavenging activity, a calibration curve was established using α-tocopherol dissolved in MNO. The DPPH radical scavenging activity (%) was calculated using Eq. (1):

1 $$\:DPPH\:radical\:scavenging\:activity\:\left(\%\right)\:=\:\frac{{A}_{0}\:-\:{A}_{1}}{{A}_{0}}\:\times\:\:100$$

where A_0_ represents the absorbance of the control containing the DPPH solution without sample, and A_1_ represents the absorbance measured after reaction with the oil sample.

### Emulsion preparation with self-emulsifying oil

An aqueous phase was prepared by dissolving 0.02% (w/w) sodium azide in distilled water to prevent microbial growth. MNO, MNO-M1, and MNO-M2 were used as oil phases, respectively. To compare the emulsification performance of MNO-M with MNO-E, oleic acid, the most abundant fatty acid in MNO-M, was added to MNO-E at a 1:3 (w/w) ratio to approximate the lipid composition of MNO-M. This mixture was also used as an oil phase.

Coarse emulsions were prepared by homogenizing the oil phase (5%, w/w) with the aqueous phase (95%, w/w) using a high-speed homogenizer (ULTRA-TURRAX model T25 digital, IKA, Staufen, Germany) operated at 12,000 rpm for 2 min. The resulting coarse emulsions were subsequently processed using a microfluidizer (Picomax MN 250 A, Micronox, Seongnam, Korea) at 55 MPa for four passes to obtain nanoemulsions.

### Stability analysis of emulsion

#### Storage temperature and pH stability

Thermal stability was evaluated by storing freshly prepared emulsions in polypropylene test tubes at 4 °C, 25 °C, and 40 °C in the dark for up to 28 days. Analyses were conducted at storage times of 0, 1, 7, 14, 21, and 28 days.

pH stability was assessed by preparing emulsions and adjusting them to pH 3, 5, 7, and 9 using 1 mol/L HCl or 1 mol/L NaOH. The adjusted samples were transferred to individual tubes and maintained at 25 °C in the dark for 24 h before analysis.

#### Confocal laser scanning microscopy

The microstructure of the samples was examined using a confocal laser scanning microscope (CLSM) system (SP8 X, Leica Microsystems, Inc., Wetzlar, Germany). For fluorescent labeling, 3 mL of each emulsion sample was treated with 20 µL of 0.1% (v/v) Nile Red solution. The stained emulsions were observed at a fluorescent excitation wavelength of 561 nm, and micrographs were acquired using a 100× oil immersion objective lens. Images were captured and analyzed using Leica Application Suite X (LAS X) (version 1.4.6, Leica Microsystems, Wetzlar, Germany; https://www.leica-microsystems.com/products/microscope-software/p/leica-las-x-ls/).

#### Particle size and ζ-potential measurement

Emulsion particle size characteristics and ζ-potential were measured by dynamic light scattering (DLS) with a Zetasizer Nano ZS90 (Malvern Instruments Ltd., Worcestershire, UK). Before conducting the analysis, 100 µL of each emulsion sample was diluted with 4,900 µL of distilled water to minimize multiple scattering effects. The mean particle size (Z-average) was calculated from the signal intensity graph. All measurements were carried out in triplicate, with each replicate consisting of 12 individual scans averaged to obtain a representative value.

### Lipid oxidation measurement of emulsion

Lipid hydroperoxides in the emulsions were quantified according to the method of Liang et al.^34^. Briefly, 0.3 mL of each emulsion was combined with 1.5 mL of an isooctane: 2-propanol solution (3:1, v/v) by vortexing for 10 s, and the procedure was repeated three times. The mixture was then centrifuged at 3400×*g* for 10 min, after which 200 µL of the upper organic phase was collected and mixed with 2.8 mL of a methanol: butanol solution (2:1, v/v). Subsequently, 15 µL of ammonium thiocyanate (3.94 mol/L) and 15 µL of an Fe^2+^ solution were added. The Fe^2+^ reagent was freshly prepared from the supernatant obtained by mixing equal volumes of 0.132 mol/L BaCl_2_ in 0.4 mol/L HCl and 0.144 mol/L FeSO_4_. After vortexing, the reaction mixture was allowed to stand at room temperature for 20 min, and absorbance was recorded at 510 nm using a UV-Vis spectrophotometer. Lipid hydroperoxide concentrations were calculated from a calibration curve generated with cumene hydroperoxide.

### Statistical analysis

All results are expressed as the mean ± standard deviation (SD) from triplicate measurements, with each experiment was conducted at least in duplicate. Statistical analyses were carried out using SPSS software version 26.0 (SPSS Inc., Chicago, IL, USA). Differences among groups were assessed by one-way analysis of variance (ANOVA), followed by Duncan’s multiple-range test for post hoc comparisons. A *p*-value < 0.05 was considered statistically significant.

## Results and discussion

### Lipid composition of self-emulsifying oils

The fermented oil fraction (MNO-M), obtained via microbial bioconversion of native macadamia nut oil (MNO) using *E. keratini* mutant EPI-7-i, exhibited significant changes in lipid composition and physicochemical properties. Native MNO exhibited a clear, pale-yellow appearance, whereas MNO-M developed a reddish coloration with a cheese-like odor. In contrast, enzymatically modified oil (MNO-E) showed only a slight increase in turbidity (data not shown). Such sensory changes are characteristic of microbial lipid metabolism and have been associated with partial glycerolysis, lipid oxidation, and the formation of minor lipid-derived metabolites, including cyclic dipeptides and oxygenated fatty acids^31,35–37^. In contrast, lipase-catalyzed modification primarily alters acylglycerol composition without generating a broad spectrum of secondary metabolites^27^.

**Fig. 1:**
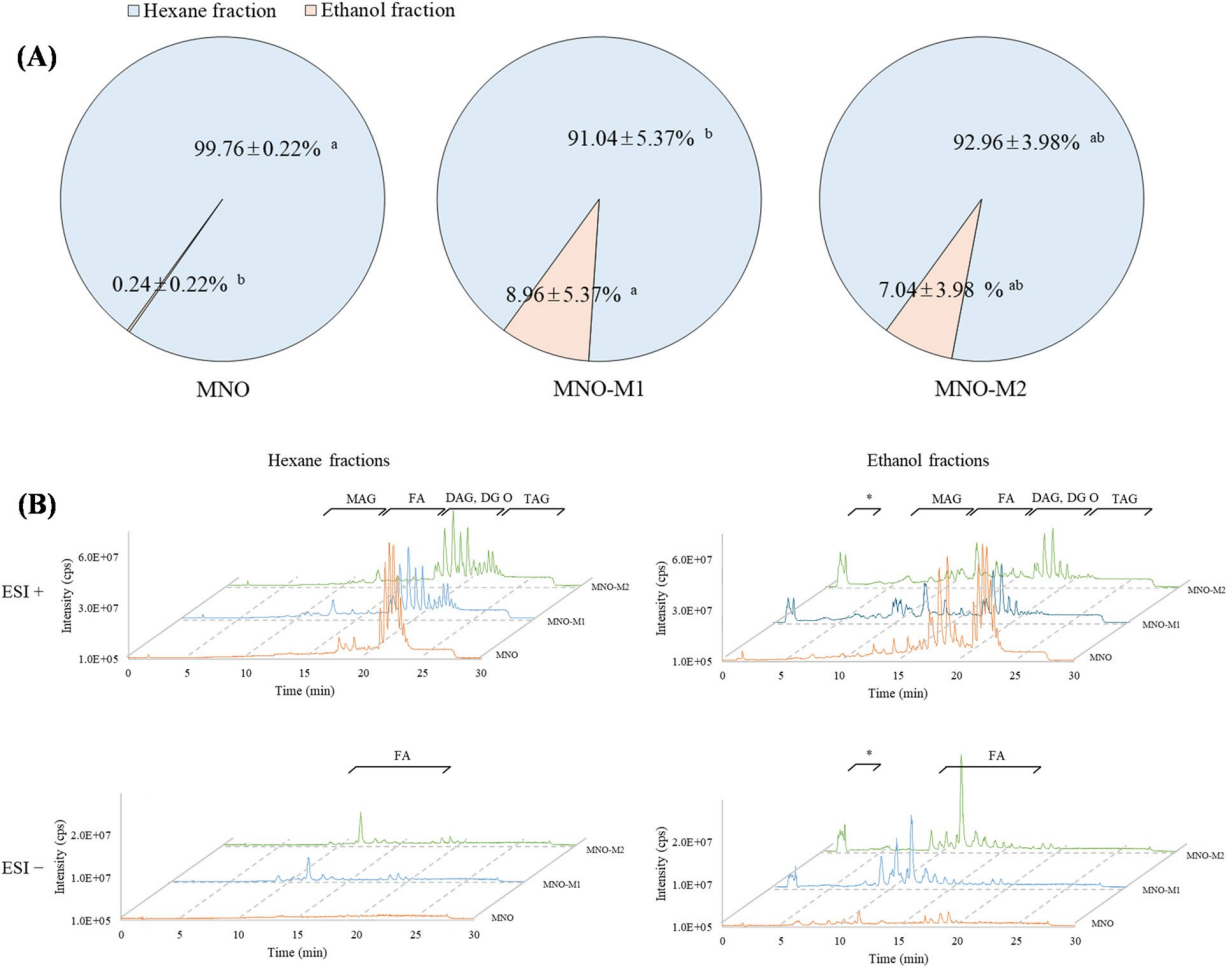
(**A**) Fractionation behavior of native macadamia nut oil (MNO) and microbially bioconverted oils (MNO-M1 and MNO-M2 after hexane–ethanol partitioning, and (**B**) total ion chromatograms of fractionated oil samples acquired in both ESI (+) and ESI (–) modes. MNO-M1 and MNO-M2 represent oils obtained after 2 and 5 days of fermentation, respectively. Lipid classes include triacylglycerols (TAG), diacylglycerols (DAG), monoacylglycerols (MAG), free fatty acids (FA), and alkylacylglycerols (DG O). Peaks marked with an asterisk (*) represent minor metabolites, including cyclic dipeptides and oxygenated fatty acids. Different letters within the same type of fractions indicate significant differences (*p* < 0.05).

To elucidate lipid compositional changes induced by microbial bioconversion, hexane–ethanol fractionation was employed to separate nonpolar and relatively polar lipid species prior to UHPLC–MS/MS analysis. All UHPLC–MS/MS analyses were performed on the fractionated oil samples rather than on the total unfractionated oils, enabling selective characterization of lipid species based on polarity. Consistent with previous studies, nonpolar triacylglycerols (TAG) preferentially partitioned into the hexane fraction, whereas monoacylglycerols (MAG), free fatty acids (FA), and other polar lipids were enriched in the ethanol fraction due to their higher polarity^28,29^. As shown in Fig. 1A, native MNO consisted almost entirely of the hexane fraction (99.76 ± 0.22%), with only trace amounts of ethanol-soluble components (0.24 ± 0.22%). In contrast, MNO-M1 and MNO-M2 exhibited significantly higher ethanol-soluble fractions, accounting for 8.96 ± 5.37% and 7.04 ± 3.98%, respectively (*p* < 0.05), indicating extensive conversion of TAG to more polar lipid species during microbial bioconversion. Notably, no further increase in the ethanol fraction was observed between MNO-M1 and MNO-M2, suggesting that the formation of polar lipids reached a quasi-equilibrium at an early stage of bioconversion.

UHPLC–MS/MS analysis further supported these findings (Fig. 1B). Native MNO was characterized by dominant TAG-related peaks at longer retention times in both ESI (+) and ESI (–) modes, whereas MNO-M1 and MNO-M2 displayed the emergence of multiple peaks at shorter retention times corresponding to MAG, FA, and other polar lipid derivatives. These changes were particularly pronounced in the ethanol fractions, confirming selective enrichment of interfacially active lipids following microbial conversion. In addition, several minor compounds, including cyclic dipeptides and oxygenated fatty acids (marked with asterisks), were detected exclusively or at higher intensities in MNO-M samples, reflecting the metabolic activity of *E. keratini* and its capacity to generate structurally diverse lipid-derived metabolites^31,35–37^. Quantitative analysis of individual lipid classes (Table S2) revealed a marked increase in MAG, DAG, and FA in MNO-M, indicative of microbial glycerolytic and oxidative pathways distinct from enzyme-driven modification^16,27,38^.

**Fig. 2:**
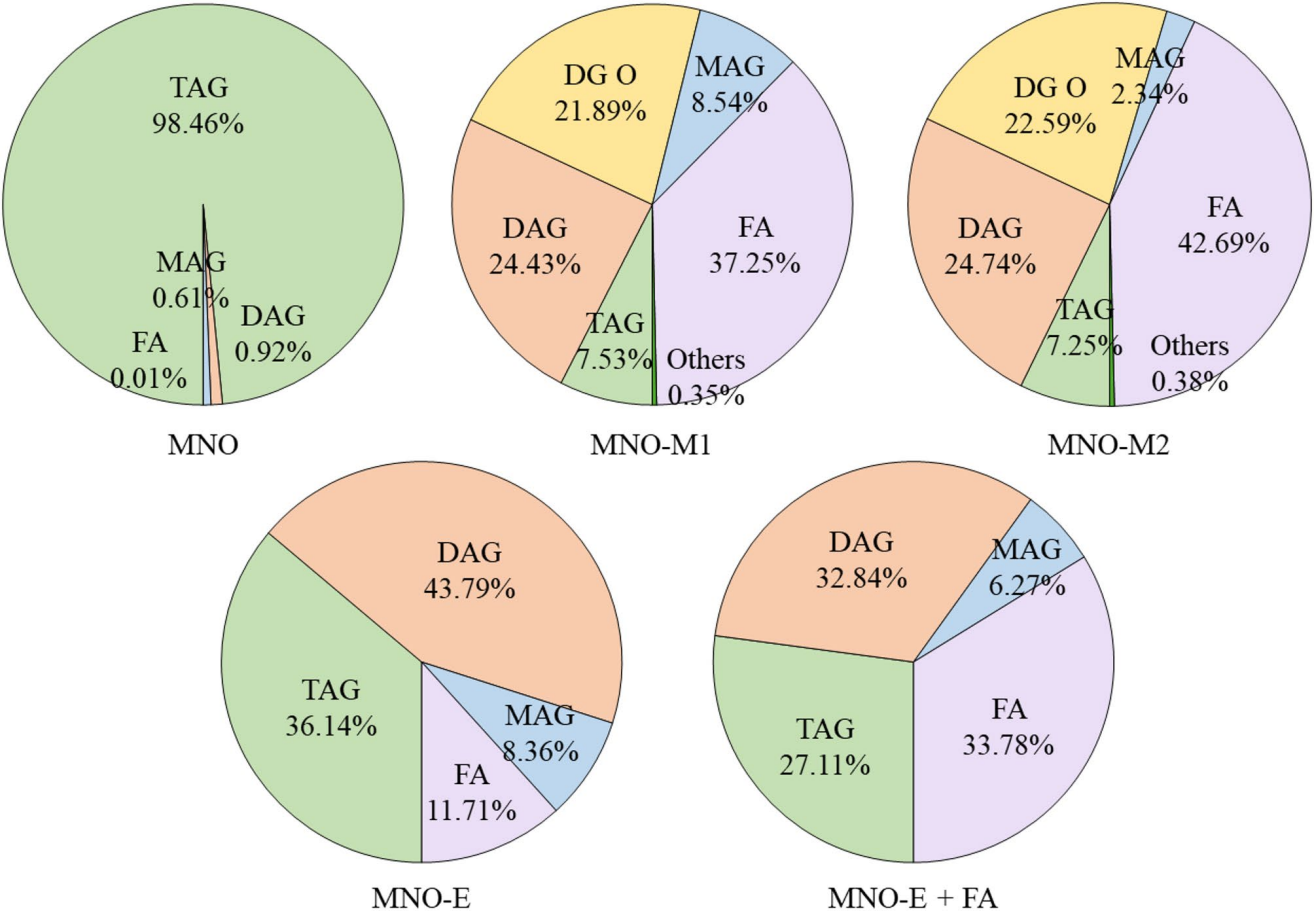
Relative weight percentages of major lipid compounds in native macadamia nut oil (MNO), microbially bioconverted oils (MNO-M1 and MNO-M2), and enzymatically modified oil (MNO-E and MNO-E + FA), determined by UHPLC–MS/MS analysis of fractionated oil samples. Lipid classes include triacylglycerols (TAG), diacylglycerols (DAG), monoacylglycerols (MAG), free fatty acids (FA), and alkylacylglycerols (DG O). MNO-E + FA represents oil with FA added to MNO-E.

Figure 2 shows the relative weight percentages of individual lipid classes in native MNO, MNO-M, and MNO-E, calculated as the sum of values ​​obtained from both solvent fractions. Microbial bioconversion by EPI-7-i resulted in profound remodeling of the lipid composition, characterized by a marked reduction in TAG and a concomitant increase in lipolytic products, including DAG, MAG, and FA. Compared with MNO, TAG content in MNO-M1 and MNO-E decreased to approximately one-tenth and one-third, respectively. This result indicates that EPI-7-i exhibits substantially higher net lipolytic activity than the commercial lipase under comparable reaction conditions.

In the broader context of lipid modification, enzymatic glycerolysis of TAG-rich oils is known to produce predictable equilibrium compositions under controlled low-water conditions, typically yielding ~ 30–35% MAG, ~ 40–50% DAG, ~ 15–25% TAG, and minimal FA (≤ 2%). This compositional framework has been shown to govern crystallization behavior and interfacial functionality, enabling the structuring of edible oils without altering fatty acid composition^27^. In the present study, MNO-E does not represent a classical glycerolysis-dominant equilibrium system. Specifically, MNO-E exhibited a relatively low MAG content (8.4%) and an elevated FA level (11.7%), alongside 43.8% DAG and 36.1% TAG. Such a composition deviates from the expected glycerolysis equilibrium and instead suggests the coexistence of hydrolysis and glycerolysis reactions. Mechanistically, lipase-catalyzed systems are governed by coupled reversible equilibria, where water activity plays a decisive role in shifting the balance between glycerolysis (favoring MAG/DAG formation) and hydrolysis (generating FA). The elevated FA level observed here indicates that hydrolytic pathways were not fully suppressed, likely due to incomplete control of water activity or reaction conditions. Consequently, the secondary glycerolysis step (DAG + glycerol → MAG) appears to have been insufficiently driven, resulting in the observed low MAG fraction. It is important to clarify that MNO-E was not intended to represent a fully equilibrated glycerolysis product, but rather to serve as a compositional reference system with reduced TAG and elevated DAG and FA levels, partially mimicking the composition of MNO-M. While the resulting composition does not perfectly match the theoretical equilibrium distribution, the increase in DAG (~ 40%) and reduction in TAG indicate that the primary objective of shifting the major lipid composition was achieved. The relatively high FA content, although unintended, likely contributed to increased surface activity of the oil phase. From an interfacial perspective, this compositional deviation has important implications. FA are more surface-active than partial glycerides and can contribute to interfacial charge through partial ionization of carboxyl groups. Therefore, the emulsification behavior of MNO-E may be influenced not only by DAG/MAG content but also by its elevated FA fraction. This aspect should be carefully considered when interpreting mechanistic differences between MNO-E and MNO-M.

Notably, compositional differences were also observed between MNO-M1 and MNO-M2. While both samples contained substantial amounts of MAG, DAG, and FA, MNO-M2 exhibited a relatively higher proportion of FA and DG O, accompanied by a slight decrease in MAG content compared to MNO-M1. This shift suggests continued lipid remodeling during extended fermentation, involving not only lipolysis but also secondary transformation pathways. Importantly, a quantitative compositional comparison between MNO-M and MNO-E indicates that both systems contain sufficient levels of surface-active lipids (DAG, MAG, and FA) to support spontaneous emulsification. In particular, DAG represents a major fraction in both oils (~ 43.8% in MNO-E and comparable levels in MNO-M), while FA is also present at substantial levels, especially in MNO-M. These overlapping compositional ranges suggest that self-emulsification in both systems may arise from partially shared interfacial mechanisms rather than fundamentally distinct ones. However, clear differences emerge in the distribution and diversity of surface-active species. MNO-E primarily consists of conventional partial glycerides generated through lipase-catalyzed glycerolysis (DAG, MAG, and FA). In contrast, MNO-M exhibits a broader composition that includes ether-linked glycerides (DG O) and various minor metabolites formed during microbial bioconversion. This indicates that microbial fermentation does not merely replicate partial glyceride generation but instead produces a more complex mixture of surface-active components. Therefore, the distinction between MNO-M and MNO-E lies not only in the presence of partial glycerides but also in the diversity and combination of surface-active molecules formed during microbial metabolism. In this context, the novelty of the microbial system is associated with the fermentation-driven generation of structurally diverse lipids and minor metabolites, rather than a fundamentally different emulsification mechanism.

The contrasting lipid profiles suggest fundamental mechanistic differences between microbial and enzymatic modification. While *C. antarctica* lipase B primarily catalyzes reversible glycerolysis reactions that can reform TAG, microbial bioconversion by EPI-7-i appears to favor net lipid degradation over re-esterification. This interpretation is supported by the higher FA content in MNO-M1 (37.25%) and MNO-M2 (42.69%) than in MNO-E (11.71%). Additionally, MAG and DAG collectively accounted for approximately 30% of total lipids in both MNO-M1 and MNO-M2. Given that MAG and DAG are well-established low-molecular-weight emulsifiers^39^, their abundance exceeds typical emulsifier-to‐oil ratios (1:3–1:10), thereby providing a clear compositional basis for the enhanced self-emulsification capacity of microbially converted oils.

Another notable feature of MNO-M was the substantial formation of alkylacylglycerols (DG O), which accounted for more than 20% of total lipids. DG O are ether lipids characterized by an ether bond at the *sn*-1 position of the glycerol backbone. In biological systems, ether lipids are more commonly found as alkyldiacylglycerols (TG O) or plasmalogens^16,38^, whereas DG O have been far less studied. TG O are abundant in marine organisms, where they contribute to buoyancy regulation and resistance to lipolytic degradation^40^, while plasmalogens are bioactive lipids implicated in cellular signaling and antioxidant defense in mammalian tissues^16^. Although ether lipids have been associated with antioxidant functions in biological systems, the present analytical approach did not provide direct structural evidence for specific functional groups, such as vinyl ether bonds. In addition, although DG O represent structurally distinct ether-linked glycerides, their emulsifying efficiency was not directly evaluated in this study. Furthermore, the chromatographic separation and structural differentiation of ether-linked lipids from conventional acylglycerols remain analytically challenging due to their similar polarity and overlapping fragmentation patterns in mass spectrometry^15,41^. Therefore, the functional role of DG O in both oxidative stability and interfacial activity remains unclear, and they should be considered as potential contributors rather than dominant functional components.

In addition to major lipid classes, several minor but potentially valuable metabolites were detected in MNO-M (Fig. 2). These included cyclic dipeptides such as Cyclo (Leu-Pro) and Cyclo (Val-Pro), which have been reported to exhibit diverse biological activities^42,43^. Dodecanedioic acid, a compound widely used as a skin-conditioning agent in cosmetic formulations^44^, was also identified. Furthermore, coproporphyrin, a reddish-brown porphyrin pigment commonly found in biological fluids^45^, was detected in microbially converted samples. MNO-M2 showed a more pronounced red coloration than MNO-M1, which corresponded with a larger coproporphyrin peak area in UHPLC-MS/MS analysis. These results suggest that coproporphyrin contributes to the characteristic red pigmentation of the bioconverted oils. Porphyrins are well-known photosensitizers that can generate singlet oxygen under light exposure, thereby potentially promoting lipid oxidation, particularly in systems rich in unsaturated fatty acids^46^. However, in complex systems such as emulsions, lipid oxidation is strongly influenced by interfacial composition and the presence of coexisting antioxidants, which can significantly modulate photosensitized oxidation processes^47^. Even though coproporphyrin levels were not quantitatively analyzed, the coproprothyrin levels detected in MNO-M were not high enough to induce lipid oxidation in MNO-M samples containing fermentation-derived bioactive compounds. As evidenced in Fig. 7, coproporphyrin was unlikely to promote significant photo-induced lipid oxidation under the tested conditions, although further studies under controlled photo-irradiation are warranted for comprehensive evaluation.

### Volatile compounds of self-emulsifying oils

**Fig. 3:**
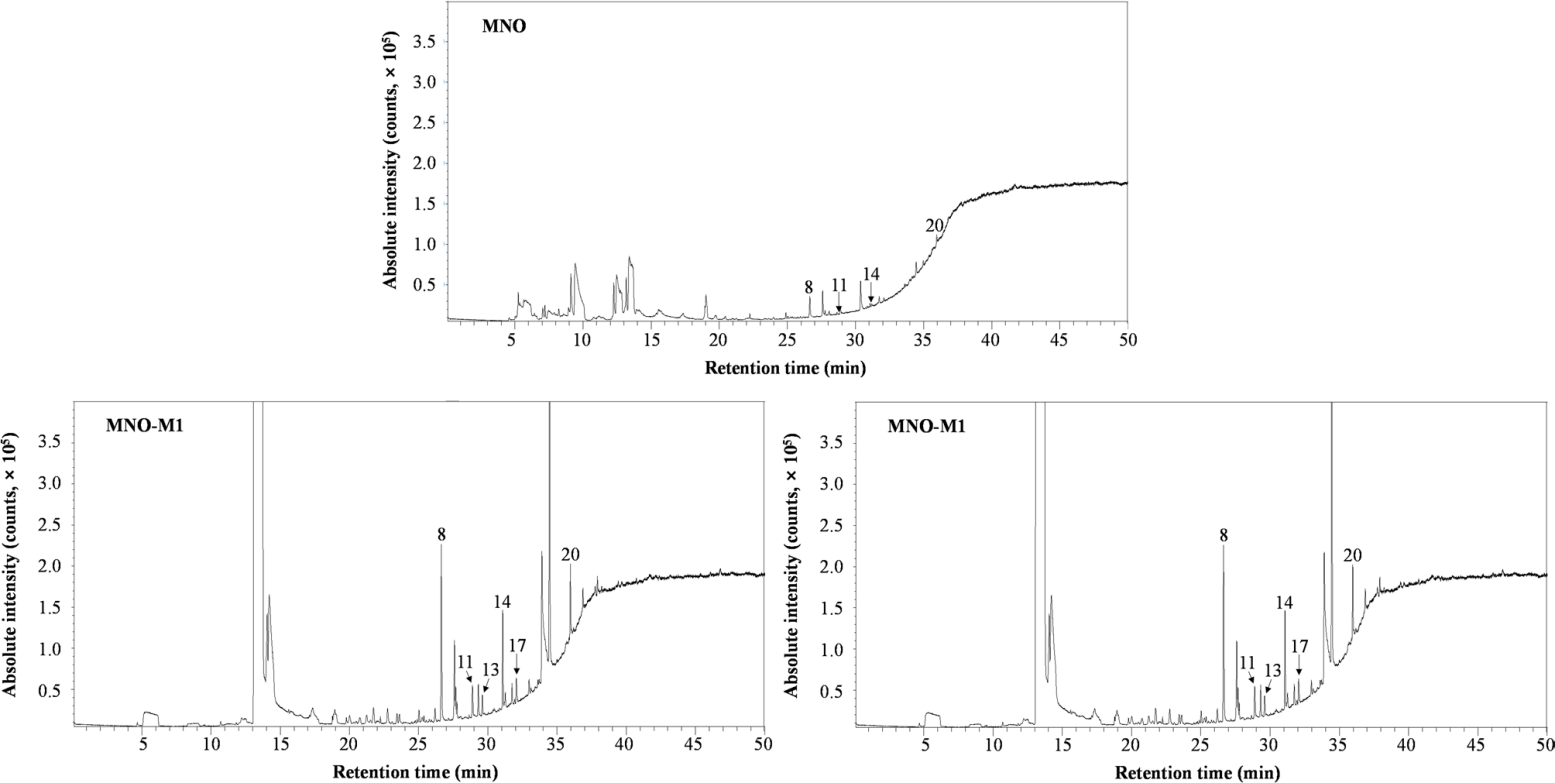
Total ion chromatograms of volatile compounds in MNO, MNO-M1, and MNO-M2 analyzed by SPME-GC-MS. Peaks are identified as follows: 8, acetic acid; 11, propanoic acid; 13, 2-methylpropanoic acid; 14, butanoic acid; 17, 3-methylbutanoic acid; 20, hexanoic acid.

The volatile compounds in native MNO and microbially converted oils (MNO-M1 and MNO-M2) were characterized using SPME-GC-MS (Fig. 3). Native MNO contained a limited set of volatiles, dominated by short-chain fatty acids (SCFAs) such as acetic acid (no. 8), propanoic acid (no. 11), butanoic acid (no. 14), and hexanoic acid (no. 20), along with minor amounts of 2-nonanone, 2-ethyl hexanol, dihydro 2(3 H)-furanone, and 2-butenoic acid. These compounds are typical lipid-derived volatiles formed through mild hydrolysis and oxidation of TAG. In contrast, MNO-M1 and MNO-M2 exhibited markedly more complex volatile profiles. In addition to the volatiles detected in native MNO, microbially converted oils contained branched-chain fatty acids, including 2-methylpropanoic acid (no. 13) and 3-methylbutanoic acid (no. 17), as well as aldehydes (e.g., 2-butenal), ketones, alcohols, esters, and nitrogen-containing heterocycles such as pyrazine, 2-methylpyrazine, 2,5-dimethyl pyrazine, and 2,6-dimethyl pyrazine. Oxygenated compounds, including 4-hydroxy-2-butanone, 1-hydroxy-2-propanone, methyl 3-hydroxybutanoate, and 1,3-butanediol, were also uniquely detected or markedly enriched in MNO-M samples.

Among these, several short-chain acids, including acetic acid (no. 8), propanoic acid (no. 11), 2-methylpropanoic acid (no. 13), butanoic acid (no. 14), 3-methyl butanoic acid (no. 17), and hexanoic acid (no. 20) are known to impart sweaty, cheesy, and pungent odors^35–37^. Quantitative comparison showed that these compounds were most abundant in MNO-M2, followed by MNO-M1, and least abundant in MNO. This trend closely mirrors the sensory observations, with odor intensity increasing in the order MNO < MNO-M1 < MNO-M2. Overall, the emergence and accumulation of diverse volatile compounds in MNO-M reflect the metabolic activity of *E. keratini* during fermentation, including amino acid catabolism, lipid hydrolysis, and secondary oxidative transformations. The production of SCFAs provides a chemical basis for the distinctive cheese-like aroma of the self-emulsifying oils, particularly in MNO-M2, and further distinguishes microbial bioconversion from enzymatic modification of MNO.

Although SCFAs are often associated with rancid or sweaty odors at high concentrations, they are also key contributors to desirable aroma profiles in many fermented foods and bio-fermented cosmetic ingredients, where they interact with esters, alcohols, ketones, and nitrogen-containing heterocycles to form complex odor matrices^16,35,36^. In particular, branched-chain acids such as 2-methylpropanoic acid and 3-methylbutanoic acid have been reported to impart characteristic fermented, nutty, or cheese-like nuances when present at moderate levels, especially in combination with pyrazines and other Maillard-type volatiles^36,37^. In this study, SCFAs produced during fermentation may contribute to off-odors. However, the volatile composition of MNO-M was dominated by a diverse mixture of acids, ketones, alcohols, and heterocyclic compounds, suggesting that the overall sensory perception arises from a balanced fermentation-derived aroma rather than from isolated SCFA-associated off-odors^48,49^. Therefore, while SCFAs may represent a potential limitation in formulation, they may also provide formulation flexibility when appropriately diluted or blended with other fragrance components, functioning as tunable contributors to “natural” or “bio-fermented” scent profiles in cosmetic applications^13,16^.

### Antioxidant activity of self-emulsifying oil

**Fig. 4:**
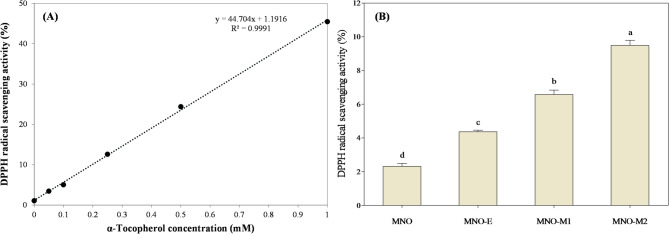
DPPH radical scavenging activity (%) of (**A**) α-tocopherol dissolved in MNO and (**B**) MNO, MNO-E, MNO‐M1, and MNO‐M2. Different letters above the bars indicate statistically significant differences (*p* < 0.05).

The antioxidant activity was evaluated by the DPPH radical scavenging assay, and the results were compared with those of α-tocopherol (Fig. 4). As estimated from the standard curve presented in Fig. 4A, the DPPH radical scavenging activities of MNO, MNO-E, MNO-M1, and MNO-M2 were equivalent to 0.02, 0.08, 0.14, and 0.22 mM α-tocopherol, respectively (Fig. 4B). Compared with native MNO, both enzymatic and microbial modifications significantly increased radical scavenging activity (*p* < 0.05), with the highest activity observed in MNO-M2. The additional antioxidant capacity generated during processing was estimated to be equivalent to approximately 0.06 mM α-tocopherol for MNO-E, 0.12 mM for MNO-M1, and 0.20 mM for MNO-M2. These results indicate that microbial bioconversion by *E. keratini* EPI-7-i was more effective than lipase-catalyzed modification in enhancing the antioxidant potential of MNO. As the DPPH assay reflects electron-donating capacity, the observed increase in radical scavenging activity suggests the formation or accumulation of antioxidant compounds capable of donating hydrogen atoms or electrons. The progressive increase in activity from MNO-M1 to MNO-M2 further indicates that these antioxidant components continued to accumulate during fermentation. Based on the compositional analysis of MNO-M, this enhancement may be attributed to the generation of FA, oxygenated lipid derivatives, ether-linked lipids, and low-molecular-weight acidic and heterocyclic compounds, several of which have been reported to exhibit antioxidant or radical-scavenging properties^45,50,51^. Therefore, these results demonstrate that microbial bioconversion of MNO not only promotes self-emulsifying behavior through lipid remodeling but also enhances its antioxidant activity. The coexistence of interfacially active lipids and antioxidant compounds in MNO-M suggests potential functional advantages for applications requiring both physicochemical stability and oxidative protection, such as cosmetic and topical formulations.

### Characterization of emulsions prepared from self-emulsifying oils

**Fig. 5:**
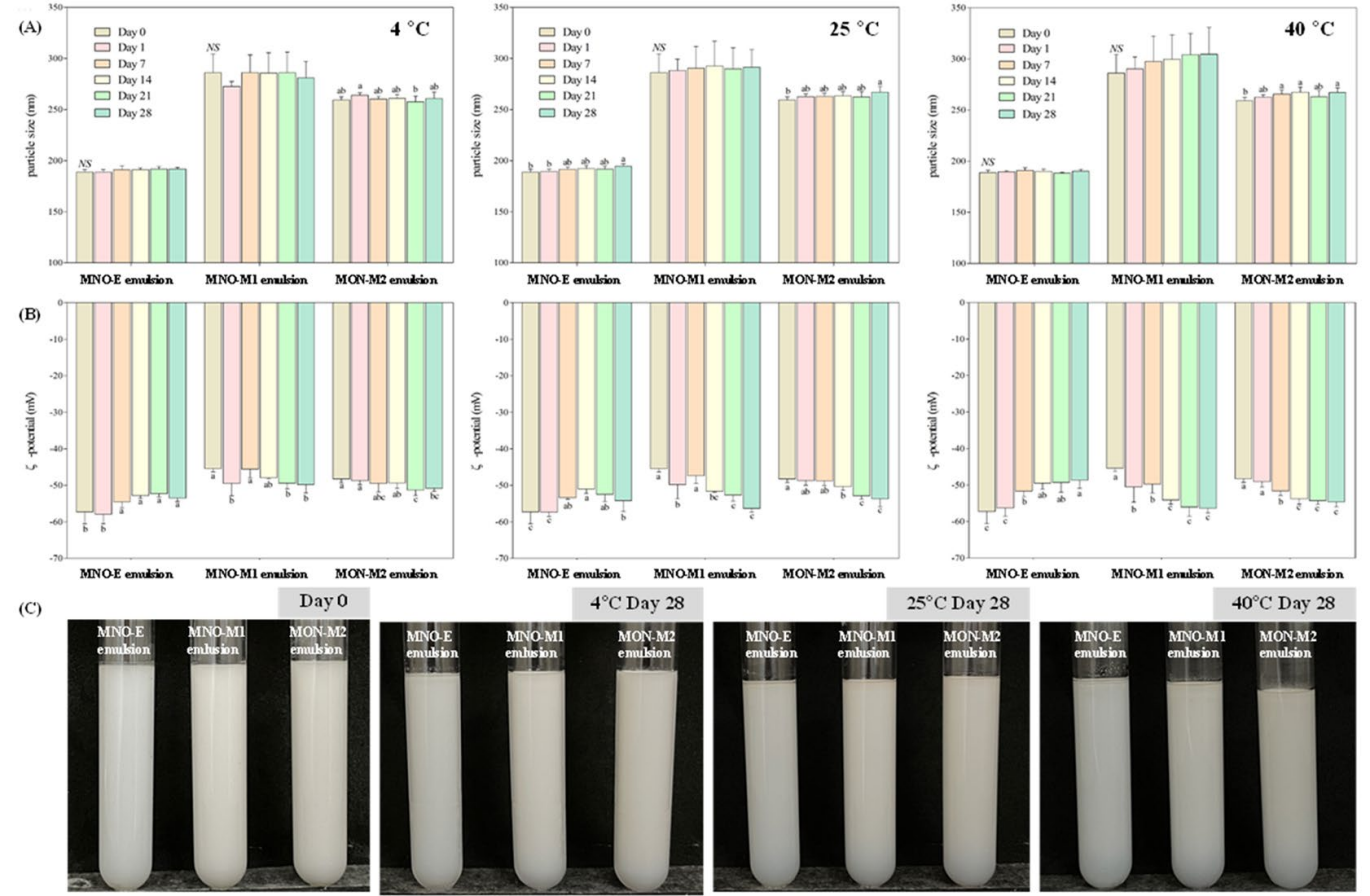
(**A**) Particle size, (**B**) ζ-potential, and (**C**) photographs of emulsions prepared with MNO‐E, MNO‐M1, and MNO‐M2 during 28 days of storage at 4 °C, 25 °C, and 40 °C. *NS* indicates no significant difference. Different letters on the graph bars within the same group denote significant differences (*p* < 0.05).

The microstructures of oil-in-water emulsions prepared from MNO-E, MNO-M1, and MNO-M2 were examined immediately after emulsification using CLSM (Fig. S1). In all samples, oil droplets were clearly visualized as red fluorescent domains following Nile Red staining, confirming successful emulsification. The droplets appeared predominantly spherical and were relatively well dispersed within the continuous aqueous phase, with no evident large-scale flocculation or coalescence observed at the initial stage. Due to the nanoscale size of the emulsions, CLSM images primarily provide qualitative information on droplet distribution rather than precise size determination^52^. Although all samples exhibited generally uniform dispersion, the limited optical resolution of CLSM prevented clear visual differentiation of relative droplet sizes among MNO-E, MNO-M1, and MNO-M2. Therefore, droplet size and size distribution were quantitatively evaluated by DLS (Fig. 5A). Immediately after emulsification, the mean droplet sizes of emulsions prepared from MNO-E, MNO-M1, and MNO-M2 were 188.78, 286.05, and 259.20 nm, respectively, confirming the formation of nanoemulsions in all systems. The corresponding ζ-potential values were − 57.27 mV for MNO-E, − 45.43 mV for MNO-M1, and − 48.27 mV for MNO-M2 (Fig. 5B). The high absolute ζ-potential values indicate strong electrostatic repulsion between droplets, which is generally associated with enhanced resistance to flocculation and coalescence^52^. The negative surface charge of these emulsions can be attributed to the presence of ionizable lipid species, such as FA and MAG, generated during enzymatic or microbial modifications of the oil. Although the MNO-E emulsion exhibited the smallest average droplet size and the highest absolute ζ-potential, the MNO-M2 emulsion achieved a comparable level of electrostatic stabilization while maintaining a similarly small droplet size. This suggests that microbial bioconversion by EPI-7-i produces a lipid composition capable of effective interfacial stabilization without the addition of external surfactants. In contrast, the relatively larger droplet size observed in MNO-M1 may reflect an intermediate stage of lipid remodeling during fermentation. Therefore, the combination of nanoscale droplet size and high absolute ζ-potential indicates that emulsions prepared from self-emulsifying oils, particularly MNO-M2, possess favorable physicochemical characteristics for physical stability. Based on these initial properties, the long-term stability of these emulsions was further evaluated under different storage times, temperatures, and pH conditions as described in the following section.

### Thermal stability of emulsions prepared from self-emulsifying oils

Nanoemulsions were prepared from self-emulsifying oils (MNO-E, MNO-M1, and MNO-M2) using water alone, without the addition of external emulsifiers, and their physicochemical stability was monitored over 28 days of storage at 4 °C, 25 °C, and 40 °C. Changes in droplet size and ζ-potential were used as primary indicators of emulsion stability, as droplet coalescence and Ostwald ripening are reflected by increases in mean droplet size over time^52^. As shown in Fig. 5A, emulsions prepared from MNO-E, MNO-M1, and MNO-M2 exhibited only minor changes in particle size throughout the storage period at all temperatures, indicating strong resistance to droplet aggregation and coalescence. No statistically significant differences (NS) were observed within each formulation during storage, even under elevated temperature conditions (40 °C), demonstrating the thermal robustness of the self-emulsifying oil systems. Among the samples, MNO-M2 consistently maintained smaller droplet sizes compared with MNO-M1, reflecting more effective interfacial stabilization following extended microbial fermentation. The high stability of these emulsions can be primarily attributed to the presence of amphiphilic lipids, including DAG, MAG, and FA (Fig. 2), which are known to exhibit higher interfacial activity than TAG due to their ability to reduce interfacial tension and promote oil–water interactions^39,53,54^. Notably, quantitative analysis (Fig. 2) revealed that although the relative proportion of MAG decreased from MNO-M1 to MNO-M2, the levels of DAG and FA were maintained or changed only slightly, resulting in a comparable overall fraction of amphiphilic lipids. This suggests that the total interfacially active lipid content remained sufficient to stabilize nanoscale droplets. Moreover, previous studies have demonstrated that mixed systems of amphiphilic lipids can form more cohesive and stable interfacial layers than systems dominated by a single emulsifier^47^. Therefore, the improved stability of MNO-M2 is likely associated with the optimized compositional balance and cooperative interfacial behavior of multiple lipid species generated during extended fermentation.

Consistent with the particle size data, the ζ-potential values of all self-emulsifying oil emulsions remained highly negative (absolute values > 45 mV) throughout storage at all temperatures (Fig. 5B). The ζ-potential reflects the surface charge of dispersed droplets and is a critical parameter governing electrostatic stabilization in colloidal systems^55^. Emulsions with absolute ζ‐potential values exceeding 40 mV are generally considered electrostatically stable^56^. The strong negative surface charge observed in these emulsions is likely derived from deprotonated carboxyl groups of FA, as well as contributions from hydroxyl-containing glycerides. Previous studies have shown that FA impart negative charges to droplet surfaces at neutral pH, thereby enhancing electrostatic repulsion^57,58^, while MAG and DAG can further reduce droplet size and increase the magnitude of negative ζ-potential^53,54^.

For comparison, emulsions prepared from unmodified MNO were also evaluated under identical conditions, although MNO did not exhibit self-emulsifying behavior. The macroscopic appearance of the emulsions during storage is shown in Figs. 5C and S2. In contrast to the self-emulsifying oils, the MNO emulsion exhibited rapid creaming within 3 days, followed by progressive phase separation over time (Fig. S2). This instability can be attributed to the absence of sufficient surface-active components capable of stabilizing the oil–water interface and preventing phase separation. The instability was particularly pronounced at 40 °C, indicating significantly reduced storage stability under elevated temperature conditions. In contrast, all self-emulsifying oil emulsions exhibited only slight creaming after approximately 7 days of storage, regardless of temperature, and remained largely homogeneous through day 28 (Fig. 5C). Creaming results from gravitational separation due to density differences between the dispersed oil droplets and the aqueous phase and does not necessarily indicate droplet growth or coalescence^51^. Although low-molecular-weight emulsifiers such as MAG and DAG do not significantly increase droplet density, the combination of small droplet sizes and strong electrostatic repulsion in these systems effectively minimized gravitational separation^59^. Overall, emulsions prepared from self-emulsifying oils demonstrated high thermal stability, characterized by minimal changes in particle size, sustained high absolute ζ-potential values, and limited creaming during prolonged storage. These results indicate the effectiveness of microbially and enzymatically modified oils as self-emulsifying systems and suggest the role of DAG, MAG, and FA in conferring intrinsic emulsion stability without the need for additional emulsifiers.

### pH stability of emulsions prepared from self-emulsifying oils

**Fig. 6:**
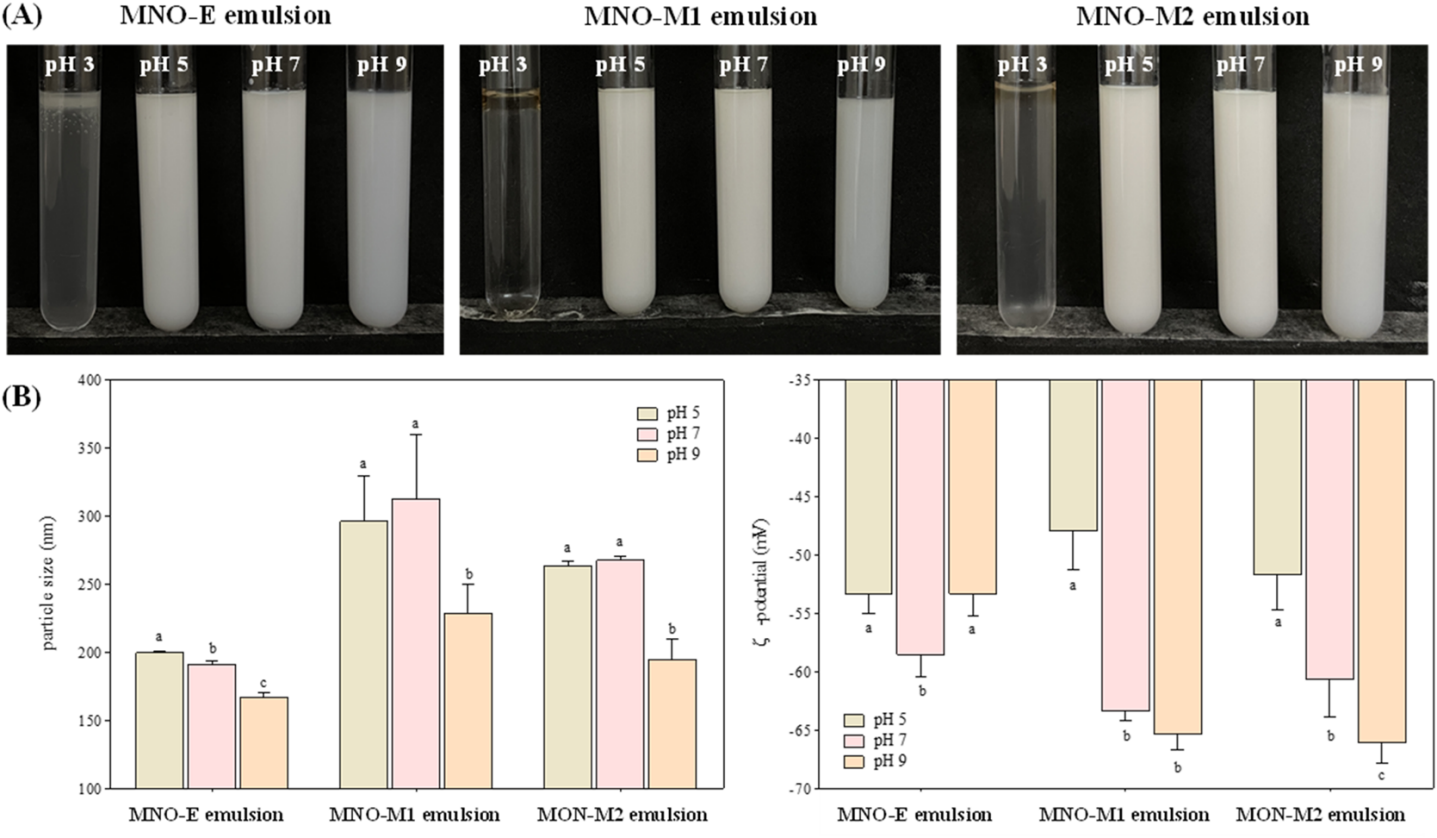
(**A**) Visual appearance, (**B**) particle size, and ζ-potential of MNO‐E, MNO‐M1, and MNO‐M2 emulsions adjusted to pH 3, 5, 7, and 9. Different letters above the bars within the sample group indicate statistically significant differences (*p* < 0.05).

The influence of pH on the stability of emulsions prepared from self-emulsifying oils was examined. As shown in Fig. 6A, emulsions remained homogeneously dispersed without visible phase separation over a pH range of 5–9. In contrast, at pH 3, a thin oil layer formed on the surface of the emulsions, indicating droplet coalescence and partial phase separation. At alkaline conditions (pH 9), MNO-M1 and MNO‐M2 emulsions exhibited highly negative ζ-potential values (approximately − 65 mV) and correspondingly small droplet sizes (Fig. 6B), reflecting strong electrostatic stabilization. As the pH decreased, the ζ‐potential values progressively shifted toward neutrality, leading to reduction in electrostatic repulsion between droplets. This decrease in repulsive forces promoted droplet aggregation, leading to increased particle sizes, particularly under acidic conditions^12,59^. The observed pH-dependent behavior can be attributed primarily to the ionization state of fatty acids present in the self-emulsifying oils. The negative surface charge of emulsion droplets arises mainly from the deprotonation of carboxylic acid groups in FA. At pH values above their pKa values, these groups exist predominantly in the anionic form (–COO−), whereas at pH values below the pKa, they remain protonated (–COOH), thereby reducing surface charge. The average surface pKa of saturated fatty acids with chain lengths from C10 to C18 is approximately 4.0^60^, whereas oleic acid, the major fatty acid in MNO, has a reported pKa of 5.02^61^. Consequently, at pH 3, most fatty acids in self-emulsifying oils are expected to be in their neutral form, leading to a substantial reduction in ζ‐potential. Under these acidic conditions, the diminished electrostatic repulsion is insufficient to counterbalance attractive forces such as van der Waals interactions, resulting in droplet aggregation and phase separation. These results suggest that the stability of self-emulsifying oil-in-water emulsions is highly pH-dependent and is governed primarily by changes in ζ-potential arising from the ionization behavior of fatty acids at the oil–water interface.

### Oxidative stability of emulsions prepared from self-emulsifying oils

**Fig. 7:**
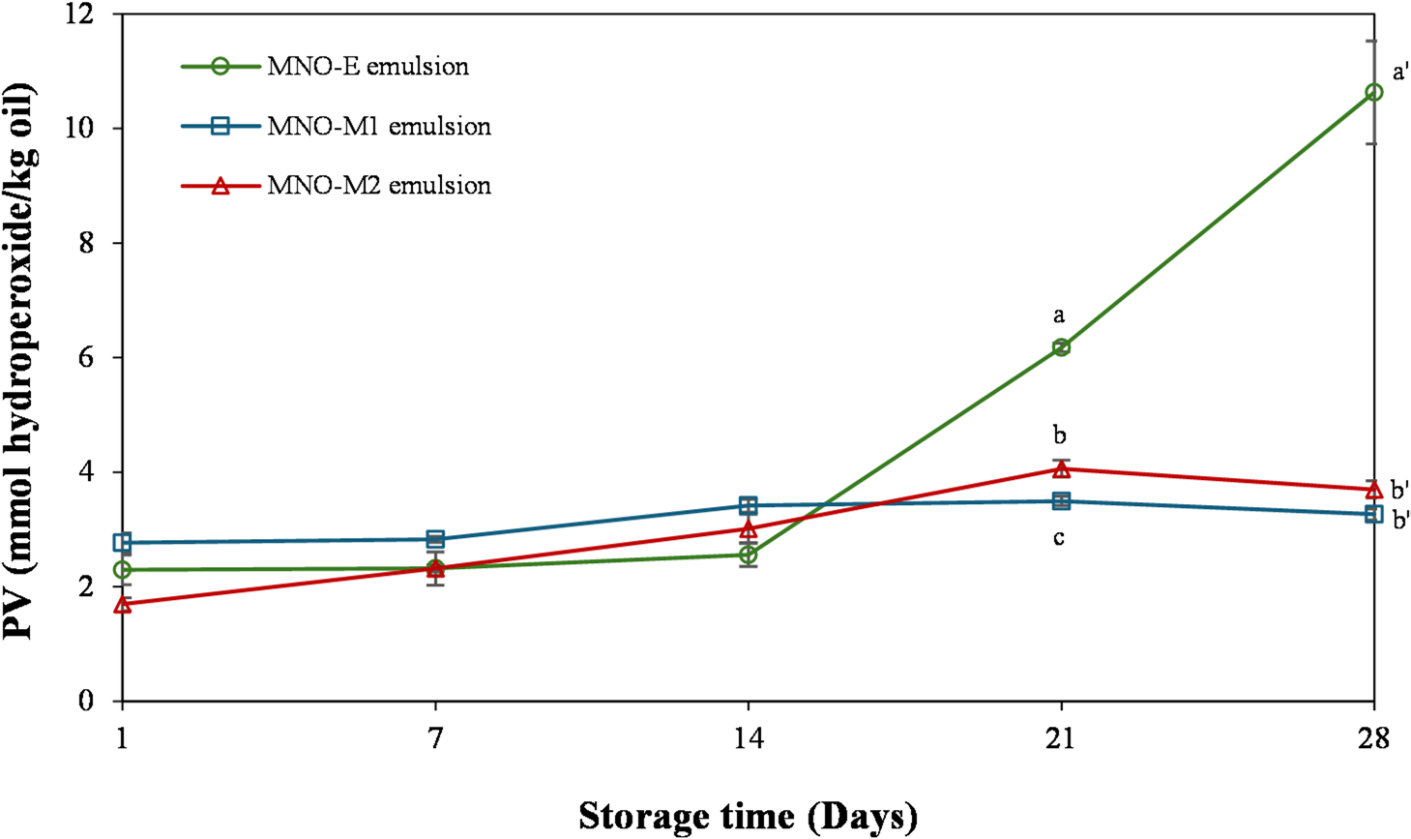
Changes in peroxide values (PV) of nanoemulsions prepared from self-emulsifying oils (MNO-E, MNO‐M1, and MNO‐M2) during 28 days of storage at 25 °C. Different letters within the same time point indicate significant differences among samples (*p* < 0.05).

The oxidative stability of emulsions prepared from self-emulsifying oils was evaluated by monitoring peroxide values (PV), which represent primary lipid oxidation products, over 28 days of storage at 25 °C (Fig. 7). All emulsions exhibited a gradual increase in PV over time, although the extent of oxidation differed depending on the emulsion system. Lipid oxidation is favored in oils rich in unsaturated fatty acids and is accelerated by initiators such as heat, metal ions, and pre-existing radicals^12^. During the first 14 days of storage, PV values remained comparable among all emulsions. However, after day 14, a notable increase in PV was observed in the MNO-E emulsion, which reached the highest level by day 28. In contrast, the MNO-M1 and MNO-M2 emulsions showed only slight and progressive increases in PV throughout the storage period, remaining below 4 mmol/kg even at the end of storage period. These results indicate that bioconverted MNO-M emulsions possess enhanced oxidative stability compared with the enzymatically treated MNO-E emulsion. In addition to PV, secondary oxidation products were further analyzed by SPME–GC–MS to provide a more comprehensive evaluation of oxidative stability. As summarized in Table S3, several oxidation-related volatile compounds, including aldehydes (e.g., heptanal), ketones, and alcohols, were detected in MNO-E. These compounds are well-known secondary products formed through the decomposition of lipid hydroperoxides^62^. Notably, aldehydes were detected only in MNO-E, whereas they were not observed in MNO-M samples, suggesting that MNO-E underwent obvious oxidative degradation. The accelerated oxidation observed in MNO-E emulsion may be partly related to its preparation process, which involved prolonged incubation at elevated temperature (65 °C for 48 h) during lipase B treatment. Thermal exposure is known to promote the decomposition of hydroperoxides into free radicals, thereby accelerating lipid oxidation^63^. Taken together, these results indicate that MNO-M emulsions exhibit improved oxidative stability possibly associated with the combined effects of microbially derived metabolites in reducing oxidative chain propagation.

Oxidative stability in emulsions is also related to interfacial properties, particularly the ζ-potential, which influences the interaction between pro-oxidant metal ions and droplet surfaces. Among the tested emulsions, MNO-E exhibited the lowest initial ζ-potential, which may have facilitated the adsorption of transition metal ions at the oil–water interface and promoted oxidative reactions^12,64^. In contrast, despite their high degree of unsaturation and strongly negative ζ-potentials, MNO-M1 and MNO-M2 emulsions exhibited limited lipid oxidation during storage. This enhanced stability can be attributed to the presence of antioxidant compounds generated during microbial bioconversion. Previous analyses demonstrated that MNO-M1 and MNO-M2 possess significantly higher antioxidant activities than MNO-E, suggesting the formation of bioactive metabolites by EPI-7-i.

The enhanced oxidative stability of MNO-M1 and MNO-M2 emulsions may be attributed to the presence of antioxidant compounds generated during microbial bioconversion. As shown in Fig. 4, bioconverted oils exhibited significantly higher antioxidant activities compared with MNO-E, suggesting the formation of bioactive metabolites by EPI-7-i. Among these metabolites, DG O (ether-linked glycerides) are of particular interest. These compounds contain ether linkages; however, the present UHPLC–MS/MS analysis does not provide direct structural evidence for the presence of vinyl ether bonds. Structurally related ether lipids, such as plasmalogens, are known to exhibit antioxidant properties by scavenging reactive oxygen species and inhibiting lipid peroxidation^16,65,66^. Therefore, the proposed functional role of DG O in enhancing oxidative stability is based on structural analogy rather than direct confirmation of neutral plasmalogen-like vinyl ether functionality. Further structural characterization using complementary techniques, such as nuclear magnetic resonance (NMR), will be required to verify the presence and specific chemical nature of ether linkages in DG O. Accordingly, the contribution of DG O should be considered as a potential, rather than definitive, mechanism.

In addition to major lipid components, several minor compounds identified by LC-MS and GC-MS may also contribute to oxidative stability. Cyclic dipeptides (e.g., cyclo (Leu-Pro), Cyclo (Val-Pro) have been reported to exhibit radical scavenging and metal-chelating activities, which may enhance oxidative resistance at the oil–water interface^67^. Pyrazine derivatives detected in MNO-M have been associated with oxidative stability in thermally processed systems, although their contribution is context-dependent^68^. Furthermore, polyols such as 1,3-butanediol may indirectly influence oxidative stability by interacting with water and reducing its effective activity, thereby limiting pro-oxidant mobility^69^. In contrast, oxygenated fatty acids and dicarboxylic acids identified in this study are more likely to represent oxidation products rather than active antioxidant species^62^.

Overall, the enhanced oxidative stability of MNO-M emulsions is likely the result of multiple contributing factors rather than a single dominant mechanism. These factors include the radical scavenging activity of microbially derived metabolites, as supported by DPPH results and reduced accessibility of pro-oxidants at the oil–water interface. Therefore, oxidative stability in this system should be interpreted as a combined outcome of compositional, interfacial, and physicochemical factors.

### Mechanistic interpretation: microbial bioconversion–driven self-emulsification and stabilization of MNO-M emulsions

**Fig. 8:**
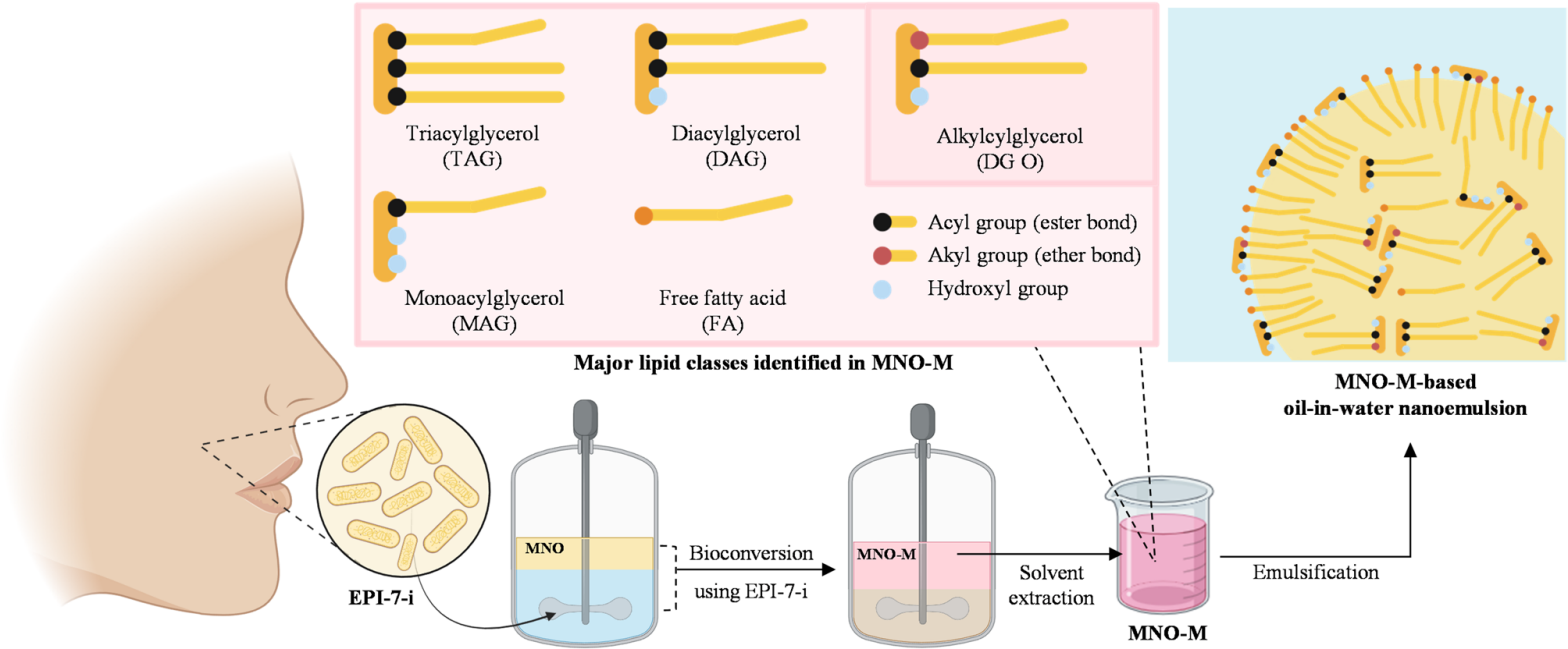
Schematic illustration of the bioconversion of macadamia nut oil (MNO) by *Epidermidibacterium keratini* mutant (EPI-7-i) and subsequent formation of an MNO-M oil-in-water (O/W) emulsion. During bioconversion, triacylglycerols (TAG) are partially converted into interfacially active lipids, including diacylglycerols (DAG), monoacylglycerols (MAG), free fatty acids (FA), and alkylacylglycerols (DG O), which promote spontaneous emulsification and contribute to the stabilization of the MNO-M emulsion.

Figure 8 schematically illustrated the bioconversion of MNO by EPI-7-i and the subsequent formation of a self-emulsifying O/W emulsion. Native MNO is primarily composed of TAG (> 90%), which exhibit inherently low interfacial activity due to their fully esterified glycerol backbone and lack of polar functional groups, resulting in poor emulsifying ability^53,54^. Microbial bioconversion by EPI-7-i promoted extensive lipid remodeling through coordinated lipolysis and etherification, yielding high levels of DAG, MAG, FA, and DG O. As schematically illustrated in Fig. 8, DAG and MAG possessed free hydroxyl groups on the glycerol backbone, which markedly enhance their amphiphilic character and enable strong adsorption at the oil–water interface, thereby reducing interfacial tension and facilitating spontaneous droplet formation^53,54,70^. Compared with TAG, DAG exhibited approximately half the interfacial tension, while MAG displayed even higher interfacial activity, making both species effective low-molecular-weight emulsifiers^39,54^. The accumulation of FA further enhanced emulsion stability by introducing ionizable carboxylic acid groups at the droplet surface. At neutral pH, partial deprotonation of these groups generated negatively charged interfaces, enhancing electrostatic repulsion between neighboring droplets and suppressing droplet aggregation^12,55^. This mechanism was consistent with the high absolute ζ-potential values (> 40 mV) measured for MNO-M emulsions, which exceeded the threshold commonly associated with stable colloidal dispersions^56^. The emulsifying performance of the enzymatically converted oil (MNO-E) supports this phenomenon that sufficiently high level of these interfacially active compounds (DAG, MAG, and FA) could enable the oil self-emulsifying, even though the extent of TAG conversion in MNO-E was relatively lower than MNO-M (Fig. 2).

Overall, while enzymatic modification produced limited quantities of interfacially active lipids, microbial bioconversion uniquely enabled the accumulation of sufficient natural emulsifiers (DAG, DG O, MAG, and FA), together with various volatile compounds and other functional produces that could contribute to antioxidant capacity. This dual generation of emulsifying and antioxidative components accounts for spontaneous emulsification, high physical stability, and enhanced resistance to lipid oxidation observed for MNO-M emulsions, without the need for external emulsifiers or synthetic antioxidants. Consequently, microbial bioconversion represents a scientifically grounded and sustainable strategy for transforming TAG-rich plant oils into multifunctional, clean-label emulsion systems for food, cosmetic, and nutraceutical applications.

## Conclusions

Microbial bioconversion of macadamia nut oil (MNO) by the skin-derived bacterium *E. keratini* mutant EPI-7-i produced intrinsically self-emulsifying oils (MNO-M1 and MNO-M2) with enhanced physicochemical and oxidative stability. The bioconversion process induced extensive lipid remodeling through coordinated lipolysis and the formation of structurally diverse lipid species, yielding higher proportions of MAG, DAG, FA, and ether-linked glycerides. These compositional features enabled the formation of stable O/W nanoemulsions characterized by nanoscale droplet sizes, high ζ-potential values, and resistance to thermal and pH stress without the need for external emulsifiers. Emulsions prepared from MNO-M oils exhibited improved oxidative stability compared with those prepared from MNO-E, suggesting the contribution of microbially derived antioxidant metabolites. However, this study has several limitations. The structural characterization of ether-linked glycerides (DG O) was based solely on UHPLC–MS/MS analysis, and the presence of specific functional groups such as vinyl ether bonds was not directly confirmed by complementary techniques (e.g., NMR spectroscopy). In addition, the quantitative analysis of lipid products, including DG O, relied on structurally similar surrogate standards, which may introduce uncertainty in absolute concentration estimates. Therefore, future studies are required to address detailed structural elucidation of ether lipids and validation of their antioxidant mechanisms under different oxidative conditions. In addition, evaluation of sensory properties, formulation compatibility, and long-term storage stability in real cosmetic systems will be necessary to support practical applications. Despite these limitations, the present study demonstrates that microbial bioconversion can serve as an effective and sustainable strategy for producing multifunctional clean-label self-emulsifying oils without the use of petroleum-based emulsifiers, suggesting their potential as next-generation ingredients for cosmetic nanoemulsion systems.

## Supplementary Information

Below is the link to the electronic supplementary material.

Supplementary Material 1

## Data Availability

Data will be made available from corresponding author on request.
