# Supplementary material for: Characterization of self-emulsifying macadamia nut oil fermented by Epidermidibacterium keratini mutant EPI-7-i originated from skin flora as a novel cosmetic ingredient
Source: Sci Rep. 2026 Apr 9;16:16186. doi: 10.1038/s41598-026-47367-z (PMC13201818; doi:10.1038/s41598-026-47367-z)
Supplement: Supplementary file 1 — Supplementary Material 1 [file 41598_2026_47367_MOESM1_ESM.docx]

**Supplementary data**

| External standard^*^ | Linear range (ppm) | Regression equation | R^2^ | Internal standard |
| --- | --- | --- | --- | --- |
| TAG 48:1 | 0.5–50.0 | y = 471160x − 162736 | 0.9994 | TAG 48:1-d7 |
| DAG 34:1 | 0.1–10.0 | y = 616468x − 77054 | 0.9986 | DAG 36:0-d5 |
| MAG 18:1 | 0.1–10.0 | y = 53700x − 2400.6 | 0.9990 | MAG 18:1-d7 |
| FA 18:1 | 2.0–50.0 | y = 74078x − 119628 | 0.9976 | FA 18:0‐d3 |
| Cyclo(Phe-Pro) | 0–0.98 | y = 163861x + 3237.5 | 0.9980 | - |
| Dodecanedioic acid | 0–4.925 | y = 21693x − 123.84 | 0.9998 | - |
| 9,10-DiHOME | 0–1.225 | y = 48248x + 363.06 | 0.9996 | - |

**Table S1.** Detailed information on lipid standards, linear ranges, and regression curves

^*^TAG; triacylglycerol; DAG, diacylglycerol; MAG, monoacylglycerol; FA, fatty acid. Each lipid is denoted by its lipid class followed by CN:DB, where CN indicates the total carbon number and DB indicates the double bond number.

**Table S2.** Concentration (mg/g) of lipid compounds in the fractionated MNO, MNO-E, MNO‐M1, and MNO‐M2

| **Compounds^*^** | **Hexane fraction** | | | |  | **Ethanol fraction** | | | |
| --- | --- | --- | --- | --- | --- | --- | --- | --- | --- |
|  | **MNO** | **MNO-E** | **MNO-M1** | **MNO-M2** |  | **MNO** | **MNO-E** | **MNO-M1** | **MNO-M2** |
| **TAG 46:2** | 3.94±0.08 | 0.74±0.02 | 0.43±0.00 | 0.42±0.00 |  | 2.17±0.03 | 0.04±0.00 | N.D.^2)^ | N.D. |
| **TAG 48:1** | 5.91±0.17 | 1.47±0.05 | 0.50±0.03 | 0.48±0.01 |  | 2.92±0.01 | 0.05±0.00 | N.D. | N.D. |
| **TAG 48:2** | 24.52±0.21 | 3.97±0.04 | 1.05±0.03 | 0.93±0.01 |  | 12.13±0.17 | 0.09±0.00 | 0.04±0.00 | 0.04±0.00 |
| **TAG 48:3** | 26.12±0.51 | 2.49±0.07 | 0.77±0.01 | 0.69±0.01 |  | 12.90±0.07 | 0.07±0.00 | N.D. | N.D. |
| **TAG 50:1** | 14.28±0.60 | 3.31±0.05 | 0.91±0.03 | 0.76±0.02 |  | 6.30±0.07 | 0.07±0.01 | N.D. | N.D. |
| **TAG 50:2** | 59.78±1.92 | 11.77±0.20 | 3.08±0.05 | 2.45±0.02 |  | 26.97±0.08 | 0.23±0.01 | 0.05±0.00 | 0.05±0.00 |
| **TAG 50:3** | 91.34±0.71 | 15.33±0.28 | 3.46±0.07 | 2.71±0.01 |  | 42.39±0.55 | 0.26±0.01 | 0.06±0.00 | 0.05±0.00 |
| **TAG 50:4** | 4.86±0.09 | 0.90±0.01 | N.D. | N.D. |  | 2.39±0.12 | N.D. | N.D. | N.D. |
| **TAG 52:1** | 19.03±0.71 | 4.16±0.18 | 1.18±0.02 | 0.94±0.03 |  | 7.51±0.13 | 0.08±0.01 | N.D. | N.D. |
| **TAG 52:2** | 85.04±1.80 | 17.25±0.52 | 4.84±0.06 | 3.97±0.15 |  | 35.00±0.82 | 0.32±0.02 | 0.06±0.00 | 0.06±0.00 |
| **TAG 52:3** | 129.94±5.86 | 29.20±0.47 | 7.53±0.12 | 5.81±0.09 |  | 56.48±0.23 | 0.51±0.01 | 0.08±0.00 | 0.08±0.00 |
| **TAG 52:4** | 12.01±0.44 | 2.72±0.10 | 0.83±0.00 | 0.68±0.01 |  | 5.32±0.18 | 0.07±0.00 | N.D. | N.D. |
| **TAG 52:5** | 1.93±0.06 | N.D. | N.D. | N.D. |  | 0.99±0.03 | N.D. | N.D. | N.D. |
| **TAG 54:1** | 18.51±0.81 | 3.43±0.08 | 1.05±0.01 | 0.88±0.03 |  | 6.43±0.09 | 0.07±0.00 | N.D. | N.D. |
| **TAG 54:2** | 66.55±2.08 | 12.17±0.29 | 3.69±0.07 | 2.93±0.14 |  | 24.19±0.52 | 0.21±0.01 | 0.05±0.00 | 0.05±0.00 |
| **TAG 54:3** | 116.07±2.92 | 23.75±0.60 | 6.83±0.09 | 5.56±0.20 |  | 45.88±1.27 | 0.43±0.01 | 0.08±0.00 | 0.08±0.00 |
| **TAG 54:4** | 10.32±0.32 | 2.61±0.08 | 0.88±0.02 | 0.73±0.01 |  | 4.43±0.08 | 0.07±0.00 | N.D. | N.D. |
| **TAG 54:5** | 2.18±0.07 | 0.67±0.00 | N.D. | N.D. |  | 1.02±0.02 | N.D. | N.D. | N.D. |
| **TAG 56:1** | 11.41±0.47 | 2.28±0.05 | 0.80±0.00 | 0.69±0.01 |  | 3.65±0.09 | N.D. | N.D. | N.D. |
| **TAG 56:2** | 37.97±0.98 | 6.47±0.14 | 2.14±0.04 | 1.74±0.06 |  | 12.40±0.22 | 0.11±0.00 | N.D. | N.D. |
| **TAG 56:3** | 24.10±0.81 | 4.81±0.08 | 1.73±0.03 | 1.43±0.06 |  | 8.97±0.19 | 0.10±0.00 | N.D. | N.D. |
| **TAG 58:1** | 4.29±0.12 | 1.23±0.02 | N.D. | N.D. |  | 1.38±0.05 | N.D. | N.D. | N.D. |
| **TAG 58:2** | 13.14±0.30 | 2.59±0.05 | 0.95±0.03 | 0.81±0.03 |  | 4.07±0.08 | 0.06±0.00 | N.D. | N.D. |
| **TAG 58:3** | 4.05±0.13 | 1.29±0.12 | 0.65±0.00 | N.D. |  | 1.90±0.06 | N.D. | N.D. | N.D. |
| **TAG 60:2** | 4.63±0.15 | 0.98±0.00 | N.D. | N.D. |  | 1.36±0.03 | N.D. | N.D. | N.D. |
| **TAG 62:2** | 1.05±0.02 | N.D. | N.D. | N.D. |  | N.D. | N.D. | N.D. | N.D. |

**Table S2** (continued)

| **Compounds** | **Hexane fraction** | | | |  | **Ethanol fraction** | | | |
| --- | --- | --- | --- | --- | --- | --- | --- | --- | --- |
|  | **MNO** | **MNO-E** | **MNO-M1** | **MNO-M2** |  | **MNO** | **MNO-E** | **MNO-M1** | **MNO-M2** |
| **DG O-31:2** | N.D. | N.D. | 0.90±0.04 | 0.78±0.01 |  | N.D. | N.D. | N.D. | N.D. |
| **DG O-33:1** | N.D. | N.D. | 1.76±0.04 | 1.58±0.11 |  | N.D. | N.D. | N.D. | N.D. |
| **DG O-33:2** | N.D. | N.D. | 7.53±0.16 | 6.50±0.25 |  | N.D. | N.D. | 0.21±0.03 | 0.13±0.01 |
| **DG O-33:3** | N.D. | N.D. | 8.57±0.34 | 7.25±0.37 |  | N.D. | N.D. | 0.22±0.03 | 0.11±0.00 |
| **DG O-35:1** | N.D. | N.D. | 2.57±0.08 | 2.30±0.07 |  | N.D. | N.D. | 0.10±0.00 | 0.09±0.00 |
| **DG O-35:2** | N.D. | N.D. | 17.07±0.67 | 14.78±0.65 |  | N.D. | N.D. | 0.44±0.01 | 0.30±0.01 |
| **DG O-35:3** | N.D. | N.D. | 30.15±0.66 | 25.63±1.35 |  | N.D. | N.D. | 0.71±0.01 | 0.40±0.00 |
| **DG O-37:1** | N.D. | N.D. | 0.97±0.01 | 0.88±0.03 |  | N.D. | N.D. | N.D. | N.D. |
| **DG O-37:2** | N.D. | N.D. | 7.40±0.25 | 6.51±0.07 |  | N.D. | N.D. | N.D. | N.D. |
| **DG O-37:3** | N.D. | N.D. | 30.88±0.37 | 26.09±1.35 |  | N.D. | N.D. | 0.60±0.05 | 0.41±0.03 |
| **DG O-37:4** | N.D. | N.D. | 2.82±0.10 | 2.29±0.08 |  | N.D. | N.D. | N.D. | N.D. |
| **DG O-39:2** | N.D. | N.D. | 5.11±0.70 | 4.18±0.64 |  | N.D. | N.D. | N.D. | 0.06±0.00 |
| **DG O-39:3** | N.D. | N.D. | 6.74±0.17 | 6.05±0.16 |  | N.D. | N.D. | N.D. | N.D. |
| **DG O-41:2** | N.D. | N.D. | 2.04±0.03 | 1.88±0.13 |  | N.D. | N.D. | N.D. | N.D. |
| **DG O-41:3** | N.D. | N.D. | 1.25±0.02 | 1.09±0.02 |  | N.D. | N.D. | N.D. | N.D. |
| **DAG 30:1** | N.D. | 0.61±0.00 | N.D. | N.D. |  | 0.65±0.01 | 0.30±0.00 | 0.08±0.00 | N.D. |
| **DAG 32:1** | N.D. | 5.99±0.43 | 3.96±0.14 | 3.26±0.18 |  | 5.44±0.14 | 2.07±0.02 | 0.59±0.01 | 0.26±0.02 |
| **DAG 32:2** | N.D. | 9.16±0.14 | 6.52±0.28 | 5.29±0.16 |  | 11.99±0.25 | 4.90±0.04 | 1.34±0.02 | 0.54±0.02 |
| **DAG 34:1** | 1.10±0.04 | 16.80±0.32 | 13.22±0.56 | 11.35±0.66 |  | 13.85±0.19 | 4.48±0.08 | 1.54±0.04 | 0.78±0.01 |
| **DAG 34:2** | 1.81±0.04 | 46.47±0.39 | 42.39±1.55 | 36.36±1.78 |  | 42.53±0.81 | 14.63±0.24 | 5.67±0.09 | 2.59±0.06 |
| **DAG 34:3** | N.D. | 1.80±0.02 | 1.40±0.06 | 1.04±0.03 |  | 2.69±0.04 | 1.06±0.01 | 0.24±0.00 | 0.10±0.00 |
| **DAG 34:4** | N.D. | N.D. | N.D. | N.D. |  | 1.07±0.01 | 0.14±0.00 | N.D. | N.D. |
| **DAG 36:1** | N.D. | 12.92±0.16 | N.D. | N.D. |  | N.D. | 2.90±0.62 | N.D. | N.D. |
| **DAG 36:2** | 4.10±0.25 | 61.33±0.68 | 59.21±2.64 | 50.57±2.61 |  | 52.37±0.78 | 15.75±0.14 | 6.12±0.10 | 2.81±0.20 |
| **DAG 36:3** | N.D. | 5.52±0.03 | 4.55±0.19 | 3.39±0.01 |  | 8.38±0.14 | 2.12±0.02 | 0.56±0.00 | 0.21±0.00 |
| **DAG 36:4** | N.D. | 0.50±0.01 | N.D. | N.D. |  | 0.92±0.01 | 0.22±0.00 | 0.06±0.00 | N.D. |
| **DAG 38:1** | N.D. | 7.33±0.04 | 4.33±0.07 | 3.60±0.09 |  | 1.60±0.02 | 1.97±0.03 | 0.33±0.01 | 0.16±0.01 |
| **DAG 40:1** | N.D. | 2.50±0.09 | 1.46±0.05 | 1.24±0.03 |  | N.D. | 0.58±0.01 | 0.08±0.00 | N.D. |
| **DAG 40:2** | N.D. | 1.66±0.03 | 0.91±0.02 | 0.77±0.02 |  | N.D. | 0.38±0.00 | 0.08±0.00 | N.D. |
| **DAG 42:1** | N.D. | 1.01±0.01 | 0.71±0.01 | 0.61±0.01 |  | N.D. | 0.18±0.00 | N.D. | N.D. |

**Table S2** (continued)

| **Compounds** | **Hexane fraction** | | | |  | **Ethanol fraction** | | | |
| --- | --- | --- | --- | --- | --- | --- | --- | --- | --- |
|  | **MNO** | **MNO-E** | **MNO-M1** | **MNO-M2** |  | **MNO** | **MNO-E** | **MNO-M1** | **MNO-M2** |
| **MAG 16:0** | 4.82±0.11 | 3.94±0.29 | 3.36±0.57 | 3.62±0.24 |  | 5.47±0.87 | 1.81±0.16 | 0.83±0.21 | 0.29±0.04 |
| **MAG 16:1** | N.D. | N.D. | N.D. | N.D. |  | N.D. | 10.58±0.82 | 8.11±1.43 | N.D. |
| **MAG 18:0** | N.D. | N.D. | N.D. | N.D. |  | N.D. | N.D. | 1.30±0.18 | N.D. |
| **MAG 18:1** | N.D. | 19.60±1.30 | 33.86±0.35 | 5.59±0.31 |  | 17.97±1.58 | 24.88±2.18 | 57.89±7.30 | 9.59±1.14 |
| **MAG 18:2** | N.D. | N.D. | N.D. | N.D. |  | N.D. | 1.80±0.10 | 3.98±0.54 | N.D. |
| **MAG 19:1** | N.D. | N.D. | N.D. | N.D. |  | N.D. | N.D. | 7.03±1.03 | 2.55±0.37 |
| **MAG 19:2** | N.D. | N.D. | N.D. | N.D. |  | N.D. | N.D. | 17.46±2.74 | 4.32±0.61 |
| **MAG 20:0** | N.D. | N.D. | N.D. | N.D. |  | N.D. | 0.74±0.04 | N.D. | N.D. |
| **MAG 20:1** | N.D. | N.D. | N.D. | N.D. |  | N.D. | 1.09±0.12 | N.D. | N.D. |
| **MAG 21:0** | N.D. | N.D. | N.D. | N.D. |  | N.D. | N.D. | 2.35±0.33 | 0.56±0.08 |
| **MAG 21:1** | N.D. | N.D. | N.D. | N.D. |  | N.D. | N.D. | 1.43±0.21 | 0.77±0.11 |
| **MAG 21:2** | N.D. | N.D. | N.D. | N.D. |  | N.D. | N.D. | 2.59±0.40 | 1.32±0.16 |
| **FA 16:0** | N.D. | N.D. | 12.27±0.07 | 11.44±1.83 |  | 2.89±0.19 | 6.84±0.45 | 7.79±0.23 | 4.40±0.36 |
| **FA 16:1** | N.D. | N.D. | 35.82±1.37 | 15.13±0.64 |  | 7.00±0.22 | 28.23±0.43 | 29.26±3.05 | 9.89±0.42 |
| **FA 18:0** | N.D. | N.D. | N.D. | N.D. |  | N.D. | 1.68±0.27 | 1.35±0.06 | N.D. |
| **FA 18:1** | N.D. | 13.86±0.24 | 137.22±6.82 | 156.30±8.74 |  | 20.81±0.88 | 71.17±2.82 | 66.53±3.79 | 55.16±1.90 |
| **FA 18:2** | N.D. | N.D. | 7.42±0.11 | 6.12±0.25 |  | N.D. | 5.02±0.20 | 5.77±0.52 | 3.28±0.11 |
| **FA 20:0** | N.D. | N.D. | N.D. | N.D. |  | N.D. | 2.07±0.08 | 0.89±0.04 | 0.89±0.04 |
| **FA 20:1** | N.D. | N.D. | 8.23±0.16 | 7.37±0.36 |  | N.D. | 4.20±0.13 | 1.98±0.08 | 1.98±0.08 |
| **Cyclo(Leu-Pro)** | N.D. | N.D. | N.D. | N.D. |  | N.D. | N.D. | 3.28±0.06 | 3.28±0.06 |
| **Cyclo(Phe-Pro)** | N.D. | N.D. | N.D. | N.D. |  | N.D. | N.D. | 0.97±0.04 | 0.97±0.04 |
| **Cyclo(Val-Pro)** | N.D. | N.D. | N.D. | N.D. |  | N.D. | N.D. | 1.22±0.23 | 1.22±0.23 |
| **Dodecanedioic acid** | N.D. | N.D. | N.D. | N.D. |  | N.D. | N.D. | 2.83±0.03 | 2.83±0.03 |
| **Tetradecanedioic acid** | N.D. | N.D. | N.D. | N.D. |  | N.D. | N.D. | 1.84±0.11 | 1.84±0.11 |
| **(Z)-5,8,11-trihydroxyoctadec-9-enoic acid** | N.D. | N.D. | N.D. | N.D. |  | N.D. | N.D. | 5.25±0.37 | 5.25±0.37 |
| **(Z)-9,10-dihydroxyoctadec-12-enoic acid** | N.D. | N.D. | N.D. | N.D. |  | N.D. | N.D. | 1.79±0.14 | 1.81±0.04 |

^*^ TAG, triacylglycerol; DAG, diacylglycerol; DG O, alkylacylglycerol; MAG, monoacylglycerol; FA, fatty acid. N.D. means not detected. All lipid species are represented by lipid class and CN:DB notation, where CN indicates the total carbon number and DB indicates the double bond number. Data are expressed as means ± standard deviations.

**Table S3.** Secondary oxidation-related volatile components generated from MNO-E analyzed by SPME-GC-MS.

| **Retention time (min)** | **Peak area** | **Compound name** | **Chemical class** | **Relevance to lipid Oxidation [62]** |
| --- | --- | --- | --- | --- |
| 6.64 | 899,646 | Heptanal | Aldehyde | Typical secondary oxidation product of unsaturated fatty acids |
| 7.95 | 227,510 | 1-(2-Methyl-1-cyclopenten-1-yl)ethanone | Ketone | Derived from oxidative degradation of lipid hydroperoxides |
| 8.04 | 219,926 | 3-Acetyl-1-cyclohexene | Ketone | Associated with advanced lipid oxidation pathways |
| 21.69 | 549,349 | 1,4,4-Trimethyl-2-cyclohexen-1-ol | Alcohol (terpenoid-like) | Secondary oxidation product from terpene/lipid oxidation |

**Fig. S1.** Confocal laser scanning microscopy (CLSM) images of MNO‐E, MNO‐M1, and MNO‐M2 emulsions (Magnification = 1,000×, scale bar = 5 μm). Red and black indicate oil droplets stained by Nile red and aqueous phase, respectively.

**Fig. S2.** Appearance of phase separation in nanoemulsions prepared with MNO after storage at 4 °C, 25 °C, and 40 °C.
